# Identification of potent inhibitors of NEK7 protein using a comprehensive computational approach

**DOI:** 10.1038/s41598-022-10253-5

**Published:** 2022-04-18

**Authors:** Mubashir Aziz, Syeda Abida Ejaz, Nissren Tamam, Farhan Siddique, Naheed Riaz, Faizan Abul Qais, Samir Chtita, Jamshed Iqbal

**Affiliations:** 1 Department of Pharmaceutical Chemistry, Faculty of Pharmacy, The Islamia University of Bahawalpur, Bahawalpur, 63100 Pakistan; 2 Department of Physics, College of Science, Princess Nourah bint Abdulrahman University, P.O Box 84428, Riyadh, 11671 Saudi Arabia; 3 Laboratory of Organic Electronics, Department of Science and Technology, Linköping University, 60174 Norrköping, Sweden; 4 Department of Pharmacy, Royal Institute of Medical Sciences (RIMS), Multan, 60000 Pakistan; 5 Department of Chemistry, Baghdad-Ul-Jadeed Campus, The Islamia University of Bahawalpur, Bahawalpur, 63100 Pakistan; 6 Department of Agricultural Microbiology, Faculty of Agricultural Sciences, Aligarh Muslim University, Aligarh, UP 202002 India; 7 Laboratory of Physical Chemistry of Materials, Faculty of Sciences Ben M’Sik, Hassan II University of Casablanca, Sidi Othmane, BP7955 Casablanca, Morocco; 8 Centre for Advanced Drug Research, COMSATS University Islamabad, Abbottabad Campus, Abbotabad, Pakistan

**Keywords:** Computational biology and bioinformatics, Drug discovery, Chemistry

## Abstract

NIMA related Kinases (NEK7) plays an important role in spindle assembly and mitotic division of the cell. Over expression of NEK7 leads to the progression of different cancers and associated malignancies. It is becoming the next wave of targets for the development of selective and potent anti-cancerous agents. The current study is the first comprehensive computational approach to identify potent inhibitors of NEK7 protein. For this purpose, previously identified anti-inflammatory compound i.e., Phenylcarbamoylpiperidine-1,2,4-triazole amide derivatives by our own group were selected for their anti-cancer potential via detailed Computational studies. Initially, the density functional theory (DFT) calculations were carried out using Gaussian 09 software which provided information about the compounds' stability and reactivity. Furthermore, Autodock suite and Molecular Operating Environment (MOE) software’s were used to dock the ligand database into the active pocket of the NEK7 protein. Both software performances were compared in terms of sampling power and scoring power. During the analysis, Autodock results were found to be more reproducible, implying that this software outperforms the MOE. The majority of the compounds, including **M7**, and **M12** showed excellent binding energies and formed stable protein–ligand complexes with docking scores of − 29.66 kJ/mol and − 31.38 kJ/mol, respectively. The results were validated by molecular dynamics simulation studies where the stability and conformational transformation of the best protein–ligand complex were justified on the basis of RMSD and RMSF trajectory analysis. The drug likeness properties and toxicity profile of all compounds were determined by ADMETlab 2.0. Furthermore, the anticancer potential of the potent compounds were confirmed by cell viability (MTT) assay. This study suggested that selected compounds can be further investigated at molecular level and evaluated for cancer treatment and associated malignancies.

## Introduction

Cancer is defined as an uncontrolled cell growth and is characterized by rapid proliferation of aberrant cells that extend beyond their normal bounds and infiltrate adjacent tissues and organs, resulting in metastasis^1^. According to the World Health Organization (WHO)^2^ cancer is the second biggest cause of mortality worldwide, accounting for 10 million deaths each year. Cancer is responsible for one out of every six deaths worldwide^3^. Breast cancer (685,000 deaths), liver cancer (830,000 deaths), stomach cancer (769,000 deaths), lung cancer (1.80 million deaths), and colon and rectum cancer (935,000 deaths) were the leading causes of cancer death in 2020^4^. Cell division fidelity is maintained by a variety of regulatory proteins, the most significant of which are kinases^5^. Protein kinases play a variety of roles in the cell cycle, checkpoint control, and cancer^6–8^. Mutations in the genetic makeup of protein kinases cause cell cycle dysregulation, which is a hallmark of neoplastic growth and plays a significant role in cancer start and progression^5–11^. Other risk factors for cancer development include age, a poor diet, exposure to toxins, and certain chronic illnesses^12^. Polo-like kinases (PLKs), cyclin-dependent kinases (CDKs), Aurora, and the Never in Mitosis (NEK) family of kinases are all examples of kinases^13–16^. These proteins are essential for cell cycle control^17^. Thus, inhibiting protein kinase can be an effective cancer treatment^18,19^. The NEK family of kinases is a conserved serine/threonine kinase family that plays a crucial function in modulating the signaling pathway of metabolic cells^20^. The NEK family consists of 11 members^21^. NEK1-11^17^ is an example. It is clear that two NIMA-related kinases, NEK6 and NEK7, play important roles in cell cycle control, signaling, differentiation, and proliferation^22,23^. Both of these proteins have a sequence identity of 85%^24^. NEK9 is another essential member of the NEK family that regulates checkpoints during cell division^25^. Furthermore, through downstream kinases NEK6 and NEK7, NEK9 is responsible for spindle assembly and centrosome separation. However, NEK7 performed critical roles during mitosis that NEK6 and NEK9 could not replace. In both humans and mice, NEK7 is the shortest member of the family, with around 302 amino acid residues^26^. NEK7 is involved in a variety of mitotic processes, including spindle assembly, centrosome placement, and cytokinesis. The functional specificity of NEK7 is also determined by its expression patterns in various cells and tissues. High-throughput transcriptome study revealed that NEK7 is prevalent in the majority of tissues where it regulates mitotic progression^9^. The most prevalent event that happens during the cell cycle is phosphorylation of the NEK7 protein. Kinases, which include serine-threonine specific kinases, protein tyrosine kinase, and sphingosine kinase, catalyze the transfer of high energy phosphate groups from ATP to protein substrate^27^. Phosphorylation of a protein substrate causes it to become more active or interact with other molecules, which causes a variety of physiological responses^28^. NEK7 phosphorylates the Kinesin 5 protein (Eg5) at SER 1033, causing centrosome separation prior to nuclear envelop collapse^29^. NEK7 promotes the nuclear envelop collapse by increasing phosphorylation of nuclear pore protein NUP98^30^. It is believed that reversible phosphorylation and dephosphorylation occur in around 50% of total protein^31^. Interference with the NEK7 protein causes unregulated phosphorylation, which leads to an increase in the number of mitotic cells, aberrant chromosomal segregation, multi-nucleation, and cell death^32–34^.

Protein kinases have been intensively studied targets over the last two decades^35,36^. They are being studied in order to produce newer anti-neoplastic drugs^36^. There are 53 FDA-approved medications in the United States that are known to have anti-cancer activity, and over 200 leads are in the pipeline for research into newer anti-cancer agents^37^, but very few FDA-approved inhibitors are known to have activity against the NIMA family of kinases. Only Dabrafenib has been reported to have potent inhibitory effect against BRAF-mutant melanoma and NRAS-mutant melanoma cell lines. Both mutant melanomas displayed significant levels of NEK9 and CDK16 protein expression. These proteins were found in charge of mutant melanomas' proliferation and survival. Dabrafenib, in particular, had considerable action against the NEK9 protein, with an IC50 value of 1–9 nM^38^. However, none of the FDA-approved inhibitors are known to have inhibitory potential against the NEK7 protein, indicating a scarcity of effective NEK7 inhibitors. We utilized Dabrafenib as a standard inhibitor because of the functional and structural similarities between NEK7 and NEK9.

**Figure 1:**
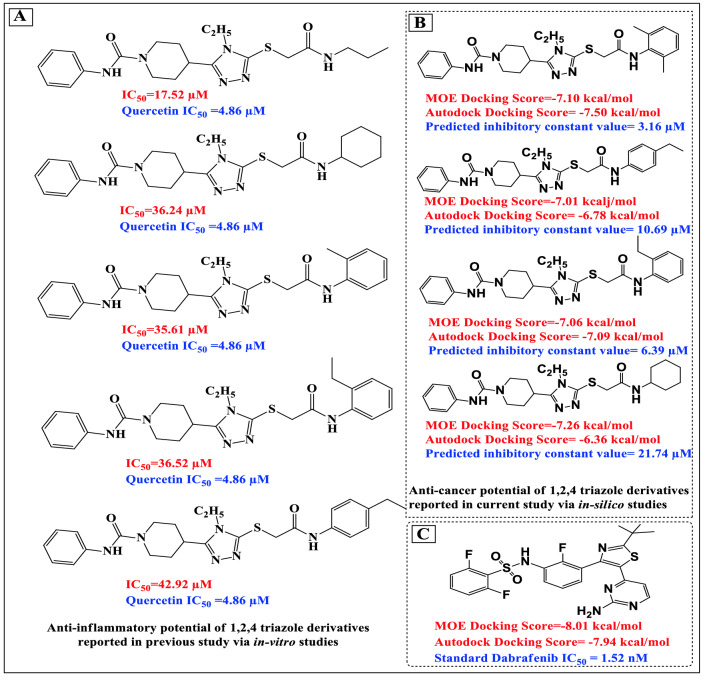
(**A**) Depiction of the most potent inhibitors of NEK7 which were previously identified as the potent inhibitors of lipoxygenase enzyme^39^. (**B**) Binding scores and binding affinity (ki) of potent derivatives against NEK7 (**C**) showing the Binding scores of Dabrafenib against NEK7: reported anti-cancer drug^38^.

This work explored the anticancer potential of novel *N*-alkyl/aralky/aryl derivatives (M1–M15) of 2-(4-phenyl-5-(1-phenylcarbamoyl) piperidine-4H-1, 2,4-triazol-3-ylthio)acetamide which have been synthesized and reported with the goal of disrupting inflammatory pathways and treating inflammatory disorders such as asthma and rheumatoid arthritis^39^. These derivatives are a heterocyclic class of compounds that interact with the receptor's active site by accepting and donating electrons and creating hydrogen bonds with the activation loop's amino acid residues. In these compounds, triazoles works as a pharmacophore and is resistant to acid–base hydrolysis, metabolic destruction, and oxidative-reductive conditions^40^. These characteristics make them attractive candidates for the creation of new anticancer agents. Additionally, different types of triazoles have been identified as antidepressant, antibacterial, anti-inflammatory, antirheumatic, anticancer, bactericidal, herbicidal, insecticidal, diuretic, and anticonvulsant^41–43^. Additionally, they exhibit a strong inhibitory effect on a variety of enzymes, including tumor-associated carbonic anhydrase, ACHE, aromatase, xanthine oxidoreductase, adenosine deaminase, and lipoxygenase^44^ (Fig. 1). The purpose of this research was to identify a new physiologically active marcapto of 1,2,4-triazole amide that might provide as a strong lead for NEK7 inhibitor development.

The approach of structure-based in-silico drug design was utilized to produce novel inhibitors of the NEK7 protein that may be employed as a powerful and specific therapy option for cancer patients. Overall, docking programs obtained a success rate of 65–70%^45^, which means that employing several docking programs and combining the findings of many docking methods provides a more accurate and comprehensive assessment of protein–ligand interaction. Additionally, various docking techniques result in more consistent and accurate conformation rankings^46,47^. To improve the accuracy of docking algorithms, the current work utilized two alternative docking programs: the academically accessible Autodock 4.2^48^ and the commercially available Molecular Operating Environment (MOE) 2015.10^49^. Both docking applications employ distinct force fields and docking methods. Additionally, the results were confirmed by using comprehensive quantum chemistry calculations, including density functional theory (DFT) calculations of frontier molecular orbitals (FMOs), global and local reactivity descriptors, and molecular electrostatic potential (MEP). Moreover, stability of protein ligand complex was determined by Molecular dynamic simulations. To determine the drug likeness and physicochemical features of compounds, detailed drug like properties, i.e. ADMET properties, were determined. This is the first complete computational analysis to advise future molecular exploration of these derivatives in order to identify an optimal candidate for therapeutic innovation in breast cancer and other linked malignancies.

## Results and discussion

### Chemistry

#### Synthesis of phenylcarbamoylpiperidine-1,2,4-triazole amide derivatives

**Figure 2:**
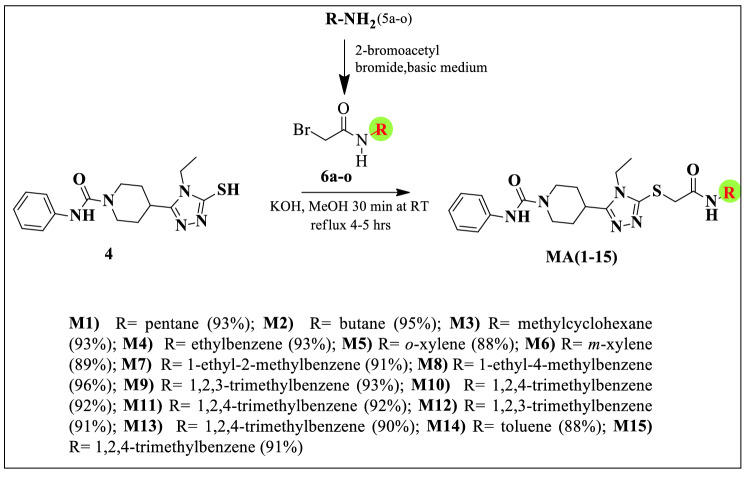
Synthesis of phenylcarbamoylpiperidine-1,2,4-triazole amide derivatives^39^.

Figure 2 depicts the principal synthesis method for the production of Phenylcarbamoylpiperidine-1,2,4-triazole amide derivatives (M1–15) which has been reported in our previous work^39^.

### Density function theory (DFT)

In current study, quantum chemical calculations have been carried out to optimize the structures of title compounds using DFT/B3LYP method with the SVP basis set implemented in Gaussian 09W^50^. All derivatives were optimized in gas and solvent (methanol) phases. The electronic properties^51^ of title compounds like E_HOMO,_ E_LUMO_ and energy gap of HOMO and LUMO was calculated. In addition, electrophilicity (**Δω±**), electronegativity(X), chemical hardness (**η**), chemical softness (S), chemical potential (**μ**) and ionization potential (eV) was also calculated. The energetic parameters of all derivatives i.e., dipole moment (Debye), polarizability (α) and optimization energy (hatree) was also calculated in gas and solvent phases as listed in Table 1. The Dipole moment is a global measure of the accuracy of the electron density of a polar molecule. It affects the interactions of a molecule with other molecules as well as electric fields. Dipole moment is the source of understanding and enumerating intermolecular interactions. The values of dipole moment for compounds M7, M8 and M12 were 3.7, 2.62 and 3.8 Debye in gas respectively and these were 4.5, 4.75 and 3.9 Debye in solvent respectively. Polarizability is chief parameter in molecular electronics which corresponds to softness of the compounds.

**Table 1:** Energetic parameters and quantum chemical descriptors for all compounds by using DFT /B3LYP/SVP method in both gas and in solvent (methanol).

Code	Gas			Methanol		
	Optimization energy (hatree)	Polarizability a.u (α)	Dipole moment (Debye)	Optimization energy (hatree)	Polarizability a.u (α)	Dipole moment (Debye)
M1	− 1729.47	307.65	4.7185	− 1733.80	386.54	4.6784
M2	− 1690.26	295.88	3.6836	− 1694.52	370.69	4.6750
M3	− 1806.52	326.91	15.220	− 1811.18	410.64	4.6946
M4	− 1846.78	325.99	4.2327	− 1846.85	423.65	11.901
M5	− 1841.83	338.56	3.0369	− 1846.84	427.11	4.3704
M6	− 1841.91	344.24	2.5975	− 1846.85	432.95	4.6478
M7	− 1881.07	350.22	3.7622	− 1886.13	441.32	4.5226
M8	− 1881.14	358.28	2.6269	− 1886.13	448.97	4.7564
M9	− 1881.07	351.64	3.6355	− 1886.13	443.31	3.5743
M10	− 1886.08	347.30	5.0251	− 1886.15	448.42	8.7084
M11	− 1881.07	352.88	2.5585	− 1886.14	444.23	4.3105
M12	− 1880.89	350.27	3.8524	− 1886.14	442.80	3.9705
M13	− 1880.96	358.78	3.7244	− 1886.14	450.77	5.0522
M14	− 1802.67	330.53	3.3577	− 1807.55	415.91	4.3180
M15	− 1886.08	339.73	4.6083	− 1886.15	443.93	6.2092
Dabrafenib	− 2407.20	319.25	6.6827	− 2407.22	434.53	9.1625

### Optimized structures

**Figure 3:**
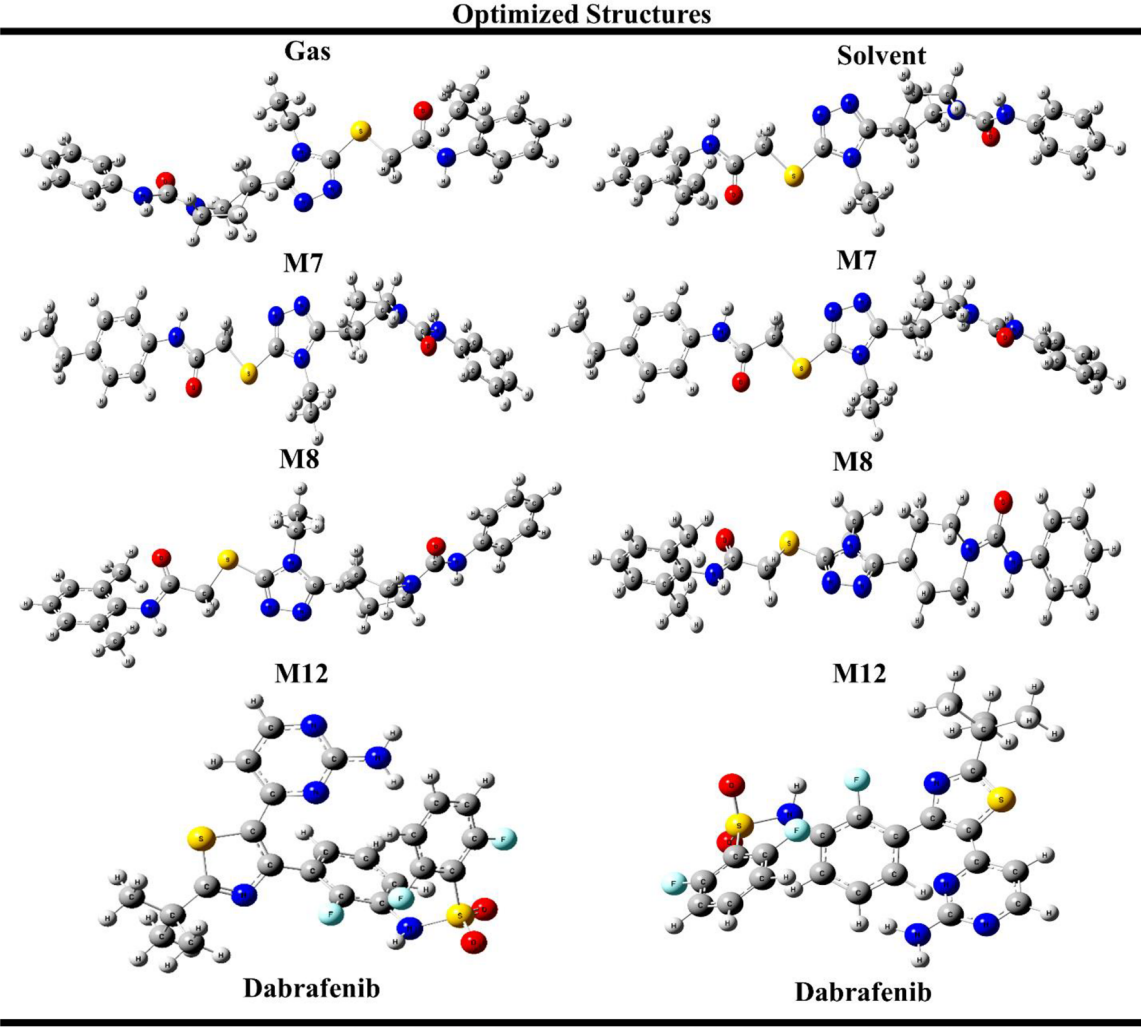
Optimized structure of potent compounds along with standard using B3LYP/SVP level of theory.

The DFT calculations of potent derivatives were performed in gas and solvent phases. The geometry of title compounds were optimized in a lowest energy singlet ground state using B3LYP/SVP level of theory. The optimized structures of potent derivatives along with standard are shown in Fig. 3.

### Frontier molecular orbital (FMOs)

The quantum chemical methods are useful in obtaining information about electro chemical behavior and molecular structures of compound. The FMOs were analyzed to obtain information about electronic and optical properties of the compounds. The energy values of highest occupied molecular orbital (HOMO) can act as electron donor whereas energy values of lowest unoccupied molecular orbital (LUMO) can act as electron acceptor. It was observed that the energies of HOMO orbitals for compound M7, M8 and M12 were same in gas and solvent phase i.e., − 0.218 eV and − 0.221 eV respectively. These energies were comparable to standard Dabrafenib which showed E_HOMO_ value of − 0.233 eV and − 0.231 eV in gas and solvent phase respectively. The E_LUMO_ for compounds M7, M8 and M12 were − 0.023, − 0.022, and − 0.019 eV in gas phase receptively. E_LUMO_ value for Dabrafenib was − 0.074 eV in gas phase. It was observed that E_LUMO_ value of compound M12 and standard Dabrafenib was relatable. The HOMO/LUMO energy gap (**∆E**_**gap**_) is an important parameter in determining molecular electrical transport properties and it showed susceptibility of compound to react with other molecules^51^. A molecule with small energy gap is more polarizable and is usually related with a high chemical reactivity and low kinetic stability. The ∆E_gap_ gap value for M7, M8, M12 and Dabrafenib was 0.194, 0.195, 0.198 and 0.159 eV in gas phase respectively. Whereas, it was 0.197, 0.195, 0.199 and 0.158 eV in solvent phase respectively. These ∆E_gap_ values indicating the relatable reactivity indices of 1,2,4 triazole acetamide derivatives with standard Dabrafenib. Moreover E_HOMO_, which is the outer orbital holding electrons, act as an electron donor and thus the ionization potential (I) is directly related to the energy of the HOMO. On the other hand, E_LUMO_ can accept electrons and the electron affinity (A) is directly related to LUMO energy. The energetic parameters of potent compounds and standard Dabrafenib are given in Table 2.

**Table 2:** Energetic parameters of M7, M8, M12 and Dabrafenib in gas and solvent phase.

Compound	E_HOMO_ (eV)	E_LUMO_ (eV)	∆E_gap_ (eV)	Potential ionization I(eV)	Affinity A(eV)	Electron donating power (ω−)	Electron accepting power (ω+)	Electrophilicity (Δω±)
**M7**								
Gas	− 0.218	− 0.023	0.194	0.218	0.023	0.147	0.027	0.174
Sol	− 0.221	− 0.023	0.197	0.221	0.023	0.150	0.027	0.177
**M8**								
Gas	− 0.218	− 0.022	0.195	0.218	0.022	0.147	0.026	0.173
Sol	− 0.220	− 0.025	0.195	0.220	0.025	0.152	0.028	0.180
**M12**								
Gas	− 0.218	− 0.019	0.198	0.218	0.019	0.143	0.024	0.168
Sol	− 0.221	− 0.021	0.199	0.221	0.021	0.147	0.026	0.172
**Dabrafenib**								
Gas	− 0.233	− 0.074	0.159	0.233	0.074	0.236	0.082	0.318
Sol	− 0.231	− 0.073	0.158	0.231	0.073	0.233	0.081	0.313

Compound	Hardness (η)	Softness (S)	Electronegativity (X)	Chemical potential (μ)	Electrophilicity index (ω)			
**M7**								
Gas	0.097	5.135	0.121	− 0.121	0.075			
Sol	0.099	5.072	0.123	− 0.123	0.076			
**M8**								
Gas	0.098	5.126	0.121	− 0.121	0.074			
Sol	0.098	5.123	0.123	− 0.123	0.078			
**M12**								
Gas	0.099	5.042	0.119	− 0.119	0.071			
Sol	0.100	5.01	0.121	− 0.121	0.074			
**Dabrafenib**								
Gas	0.080	6.28	0.154	− 0.154	0.149			
Sol	0.079	6.34	0.152	− 0.152	0.147			

Another important parameter to determine the reactivity of the compound is global reactivity descriptors. The HOMO/LUMO energies are used to determine these global reactivity parameters. The Global reactivity descriptors like molecular Hardness, Softness, Chemical potential, Electronegativity and Electrophilicity index of the potent derivatives and Dabrafenib is tabulated in Table 2. Compound M7 and M8 showed high value for chemical softness i.e., 5.1. Whereas Dabrafenib showed highest reactivity with softness value of 6.28 in gas phase.

**Figure 4:**
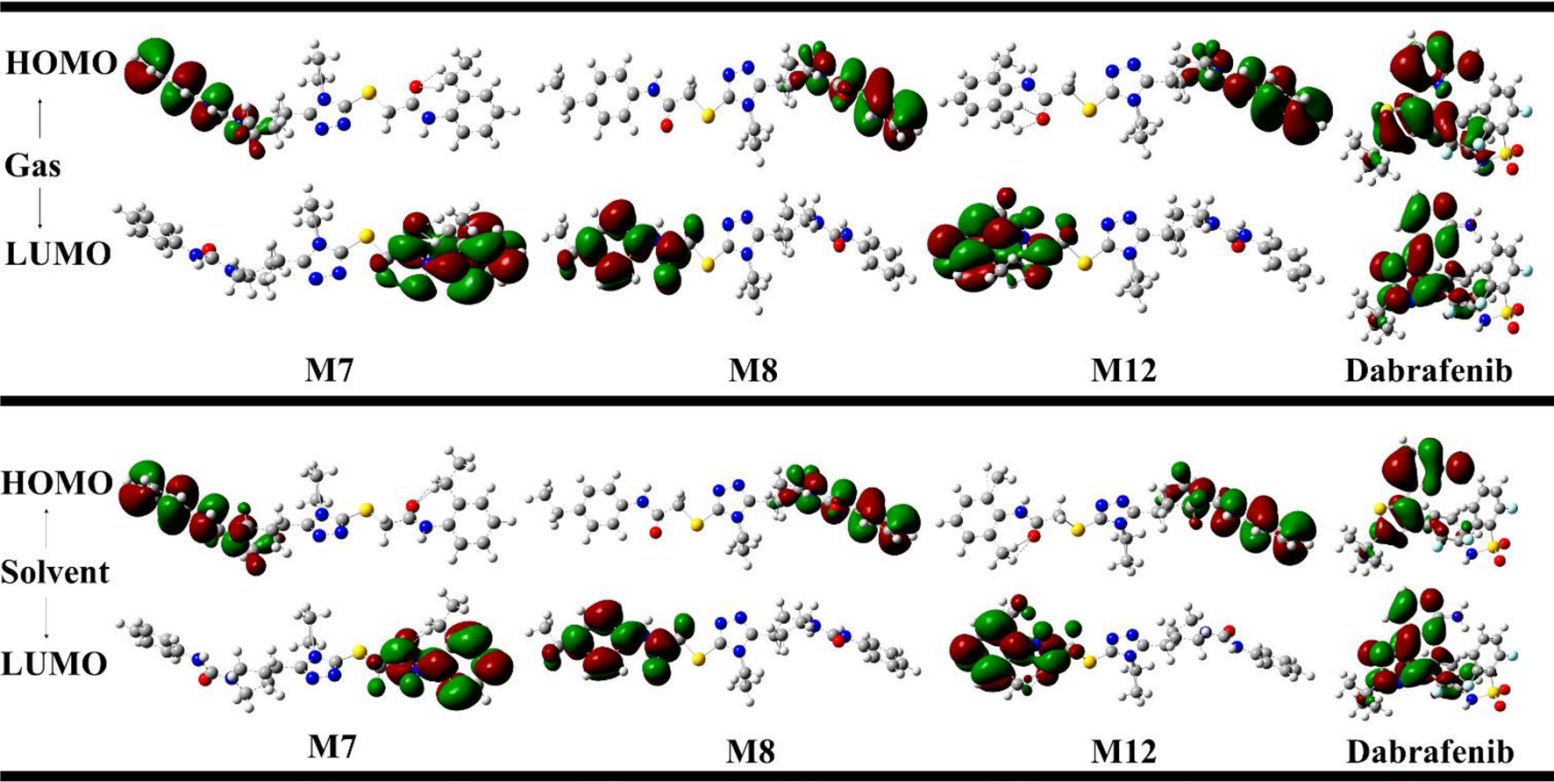
Calculated HOMO and LUMO orbitals of potent derivatives at B3LYP/SVP level of DFT calculations.

Moreover, FMOs analysis revealed that HOMO orbitals of potent derivatives in gas and solvent phases were localized to benzene ring, nitrogen atom of *N*-phenylcarbamyl part and piperdinyl ring of the compound M7, M8 and M12 (Fig. 4) which means charge transfer from HOMO to LUMO was due to contribution of π bonds and lone pairs of nitrogen atoms. Whereas, LUMO orbitals of M7 and M12 in gas and solvent phases were localized to N substituted acetamide part of compound which exhibited that electron accepting ability of compounds were due to involvement of substituted aralkyl ring.

### Molecular electrostatic potential

**Figure 5:**
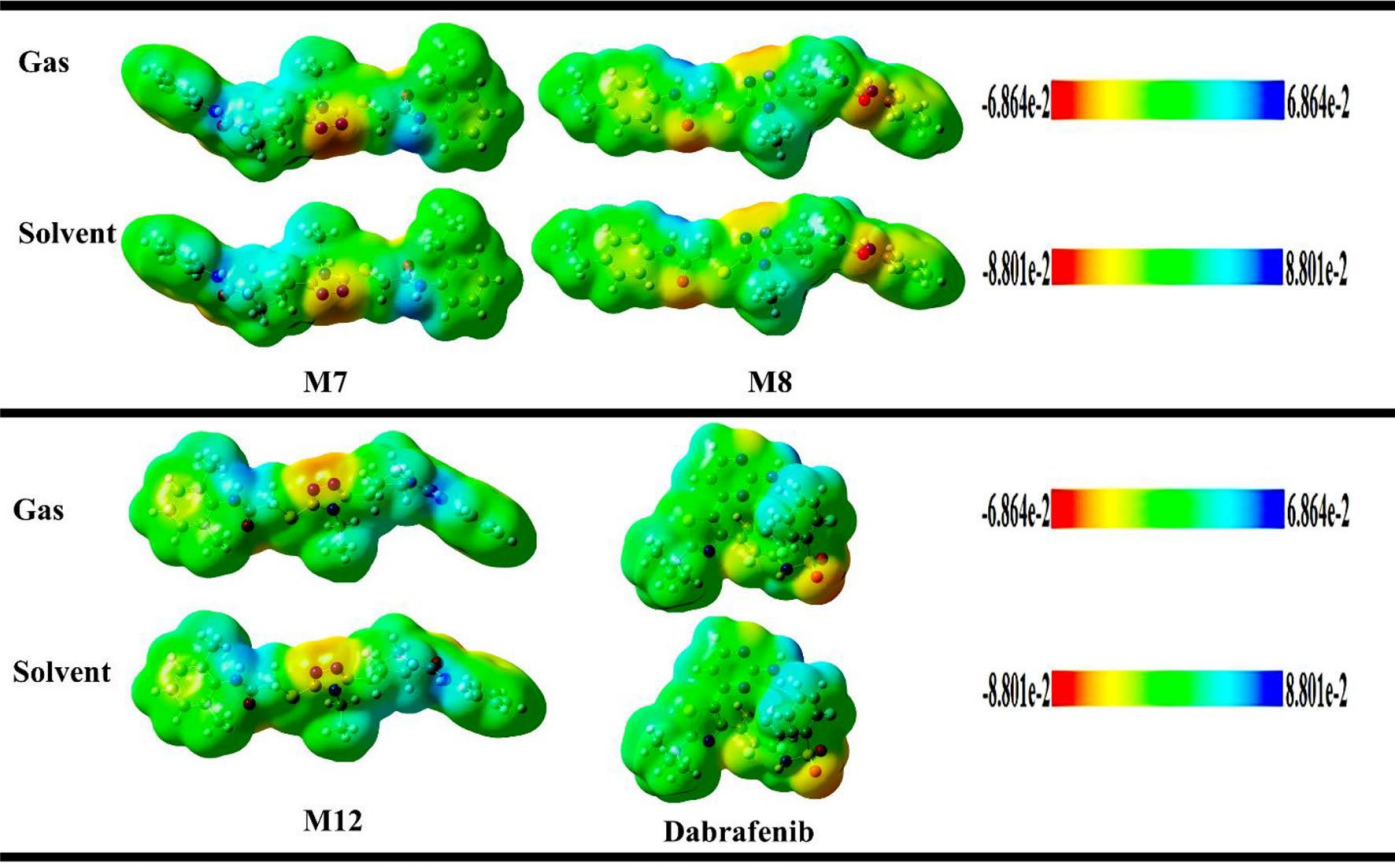
Electrostatic potential of most potent derivatives and Dabrafenib.

The MEP (molecular electrostatic potential) shows the electron density of a molecule and it is used to identify sites of positive and negative electrostatic potentials for nucleophilic and electrophilic attacks^52^. Electrostatic potential is widely used to determine distribution of electronic charge. It is very important tool to identify electrophilic and nucleophilic attack on a molecule. It is also powerful tool to identify biological recognition process and hydrogen bonding interactions. Surface and contours provide an idea about interaction of different geometries. Electrostatic potential and electron density of compounds M7, M8, M12 and Dabrafenib are shown in the Fig. 5. It can be seen that electron density is localized uniformly throughout titled molecules but as per ESP bar data, negative ESP is localized only to specific areas of a molecule. This result is obvious because ESP co-relates with partial charges and electronegativity of a molecule. Electrostatic potential values are indicated by different colors as shown in ESP bar. Highly negative electrostatic potential is represented by red color which is related to electrophilic reactivity whereas blue color is indicating highly positive potential relating to nucleophilic reactivity and green color elaborates the zero potential region^53^. It can be seen that highly negative potential was localized to triazole ring of compound M7 and M12. Whereas, compound M8 showed negative potential at oxygen atoms. These findings are important to relate electrophilic and nucleophilic reactivity of a compound.

### Molecular docking

*In-silico* molecular modeling studies are important part in drug discovery and drug development process; these studies are carried out in order to evaluate binding interactions between top ranked hits and targeted protein. So, the selected Phenylcarbamoylpiperidine-1,2,4-triazole amide derivatives were docked into active pocket of NEK7 using MOE and Autodock which evaluated their binding capacity. Among them, six compounds with binding affinity ranges from − 25.81 to − 31.38 kJ/mol had shown better and comparable binding energies in both docking software. Interestingly compounds with best ligand–protein docked complex also showed good docking scores (Table 3).

**Table 3:** Docking scores of phenylcarbamoylpiperidine-1,2,4-triazole amide derivatives.

Compound	MOE docking score (kJ/mol)	Autodock docking score (kJ/mol)	Predicted Autodock inhibitory constant value (µM), Experimental IC_50_; µM^a^
M1	− 30.45	− 25.52	33.94
M2	− 29.37	− 22.88	98.41
M3	− 30.37	− 26.61	21.74
M4	− 29.83	− 23.05	91.03
M5	− 29.74	− 26.10	26.69
M6	− 31.08	− 25.81	29.87
M7	− 29.53	− 29.66	6.39
M8	− 29.32	− 28.36	10.69
M9	− 29.99	− 25.85	29.36
M10	− 29.99	− 25.56	33.16
M11	− 28.61	− 26.98	18.81
M12	− 29.70	− 31.38	3.16
M13	− 29.87	− 26.94	18.88
M14	− 30.91	− 24.97	42.28
M15	− 29.03	− 27.94	12.69
ADP (co-crystal ligand)	− 16.40	− 13.76	3.90 (mM)
Dabrafenib (standard)	− 34.39	− 33.22	1.54 (nM)^a^

### Interpretation of protein–ligand interactions

**Figure 6:**
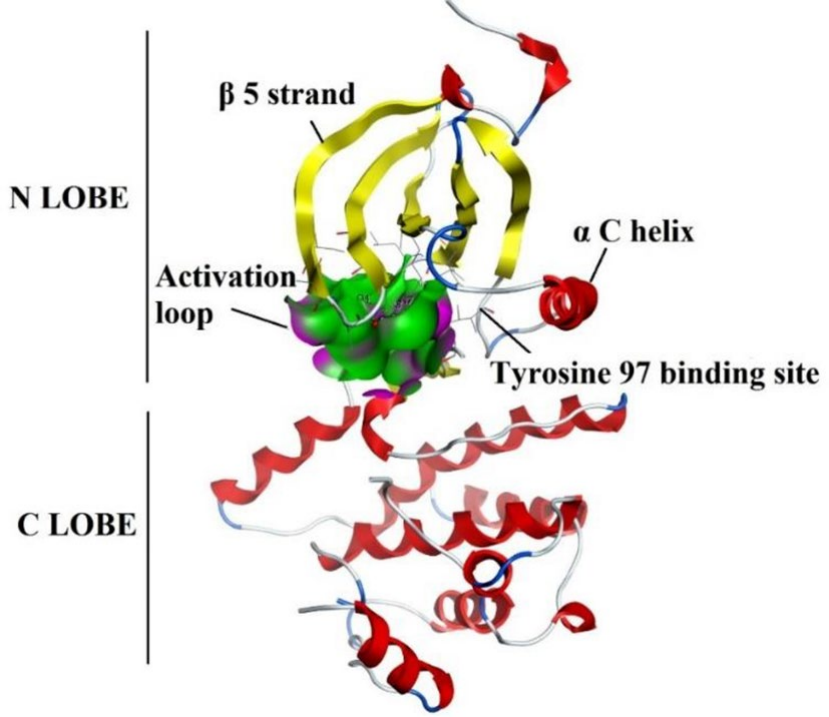
Crystal structure of NEK7 bounded to ADP; ADP bound in activation loop of NEK7 which is shown as green and pink clouds.

Commercially and academically available modelling software’s was used for docking of all compounds within active pocket of NEK7 protein. Docking results were sorted out and a compound with highest binding energy and best docking pose was selected for further interpretation. Docking protocol used in current study was validated by re-docking of co crystal ligand ADP (Fig. 6) with NEK7 protein using MOE and Autodock. ADP was observed to produce good hydrophobic and Hydrophilic interactions with important amino acid residues of activation loop i.e., LYS63, LEU111, GLU82, GLY43, SER46 and GLY112.

The Molecular docking experiment in our current study revealed that certain compounds, such as M7, M8, and M12 produced strong bonding and non-bonding interactions with amino acid residues of activation loop i.e., ALA61, ALA165, ASP118, GLY117, ALA116, ARG121, LYS63, GLY43, VAL48, GLY41, LEU111, LEU113, PHE168, ASP115, ALA114, ASP179, ILE40, ASN166, and ILE40.

Docking conformations of compound M7 revealed strong hydrophobic and hydrophilic interactions with active-site amino acid residues. Following amino acid residues were implicated in bonding and non-bonding interactions with M7; GLN44, SER46, LYS63, ILE40, ALA61, ASP115, LEU111, GLU112, ILE95, LEU113, ALA114, VAL48, ASP179, ARG42, GLY41, VAL48, PHE45, and PHE168. The Acetamide part of the compound is substituted with benzene ring bearing ethyl group at *ortho* position. The hydrophobic interactions between the substituted benzene ring and amino acid residues possessed significant importance. The benzene ring was involved in π-sigma interaction with PHE45, whereas the ethyl group at *ortho* position was exposed to van der Waals interactions with amino acid residues of active site. In addition, the ethyl group made significant contribution in stabilizing the protein–ligand complex by donating electrons and exerting a positive mesomeric effect (**+ *M***). The 1,2,4 triazolyl-3-thiol ring was engaged in π-alkyl and carbon hydrogen bonding with VAL48 and GLY41 respectively. The π-alkyl interaction contribute significantly in stabilizing the protein–ligand complexes. Furthermore, the Phenyl ring of *N*-phenylcarbamyl part was implicated in strong alkyl and π-alkyl interactions with ALA61, LEU111, and ALA114. LEU111 is a component of the protein's gate keeper residues, which maintain active conformation of the NEK7 protein. Compound M7 formed three hydrogen bonds with distinct amino acids of the active site. The acetamide part of M7 was involved in hydrogen bonding with SER46, which is the part of DFG motif. Furthermore, electronegative oxygen atom of acetamide part was forming a hydrogen bond with LYS63. Another hydrogen bonding was formed between ALA114 and the electropositive hydrogen atom of N-phenylcarbamyl component of compound M7. These hydrogen bonds have significant effect on stabilizing the protein–ligand complex. Moreover, the piperdinyl ring was involved in hydrophobic interactions with ILE40 and VAL48. Furthermore, ASP115, PHE168, ASP179, ILE95, GLU112, LEU113, GLN44, VAL65, GLY43, and ARG42 were also involved in 10 van der Waals interactions with compound M7. These interactions were playing important role in altering the protein active conformation and stabilizing the protein–ligand complex. The docking scores of MOE and Autodock was found to be − 29.56 and − 29.66 kJ/mol, respectively.

The docked conformation of compound M8 with NEK7 exhibited potent interactions with the following amino acids: LYS63, GLY41, ILE40, ALA61, ASP115, PHE168, LEU111, GLU112, ILE95, LEU113, ALA114, VAL48, ASP179, and ASN166. The N-substituted acetamide part of compound M8 has an ethyl substituted benzene ring. Ethyl group was present at *para* position of benzene ring. Although the ethyl group is an electron donor, but its substitution at the *para* position might have decreased the binding score and binding affinity of compound M8 in comparison to M7. However, ethyl group was involved in hydrophobic interactions with ALA114, ILE95 and PHE168 respectively. The benzene ring was involved in important hydrophobic interactions i.e., π–π T-shaped interaction with PHE168, π-alkyl and alkyl-alkyl interactions with ALA61, LEU113 and ILE40 respectively. Another significant interaction was π-donor hydrogen bonding was observed between benzene ring and ALA114. Moreover, It was observed that three important hydrophilic interactions were produced by M8 i.e., Carbon hydrogen bond was formed between electronegative oxygen atom of phenylcarbamyl part and LYS63 residue of active site. The electropositive hydrogen atom of acetamide and phenylcarbamyl part were involved in formation of strong hydrogen bonding with ASP115 and ASP179, respectively. Hydrogen bonding is important molecular interactions which maintain stability of the protein–ligand complex. The hydrophobic interactions were also significant in maintaining stability of the complex. Among hydrophobic interactions, it was noticed that 1,2,4 triazolyl-3-thiol ring formed π-lone pair bonding with ILE40. Whereas, piperdinyl ring of compound M8 was producing π-alkyl interaction with VAL48 residue. All these interactions ultimately disturbing the active conformation of NEK7 protein. Furthermore, 4 van der Waals interaction were also observed with LEU111, GLU112, ASN166 and GLY41. Docking score of M8 compound was found to be − 29.33 and − 28.36 kJ/mol obtained from MOE and Autodock, respectively.

Docked conformation of compound M12 showed potent interactions and formed most stable complex through bonding and non-bonding interactions with following amino acids; ALA61, ILE95, ALA114, LEU111, PHE168, VAL48, ASN166, SER46, PHE45, VAL65, CYS79, LEU180, LYS63, ASP179, ILE40, LEU113 and GLU112. Compound M12 exhibited excellent binding energy and binding affinity (***ki***) of 3.12 µM. It might be due to substitution of benzene ring along with two methyl groups at *ortho* position. It is discussed that position of alkyl froup was playing great role in determining the inhibitory potential of the compound. Two methyl groups was imparting strong mesomeric effect (**+ *M***) by donating electrons and increasing the resonance of benzene ring. It was observed that aromatic ring present in acetamide part of compound was involved in strong π-sulfur interaction with CYS79. π-sulfur interaction formed between sulfur atom of amino acid and π-electronic cloud of aromatic ring. Meanwhile same aromatic ring was also involved in π-alkyl interaction with VAL65. Second aromatic ring present in N-phenylcarbamyl part was also involved in major stabilizing electrostatic interaction. It was involved in 4 π-alkyl interactions with ALA61, ILE95, ALA114 and LEU111 respectively. Another important interaction was π-anion bonding with ASP179. It was observed that important amino acid residues of NEK7 protein was engaged in interactions by these two aromatic rings. In addition, single carbon hydrogen bond was formed between ethyl group of 1,2,4 triazolyl ring and ASP179. The triazolyl ring was also involved in two π-cation interactions with LYS63 and ASP179. The single hydrogen bond was observed between oxygen atom of acetamide part and LYS63. All these interactions were corresponding to stabilized protein–ligand complex. In addition, piperdinyl ring of M12 was producing π-alkyl interaction with VAL48 residue. Total 8 van der Waals interactions was observed with ASN166, SER46, PHE45, LEU180, PHE168, ILE40, LEU113 and GLU112. Docking scores of M12 was found to be − 29.70 and − 31.38 kJ/mol from MOE and Autodock respectively. Docking scores of compound M12 was comparable to standard Dabrafenib.

**Figure 7:**
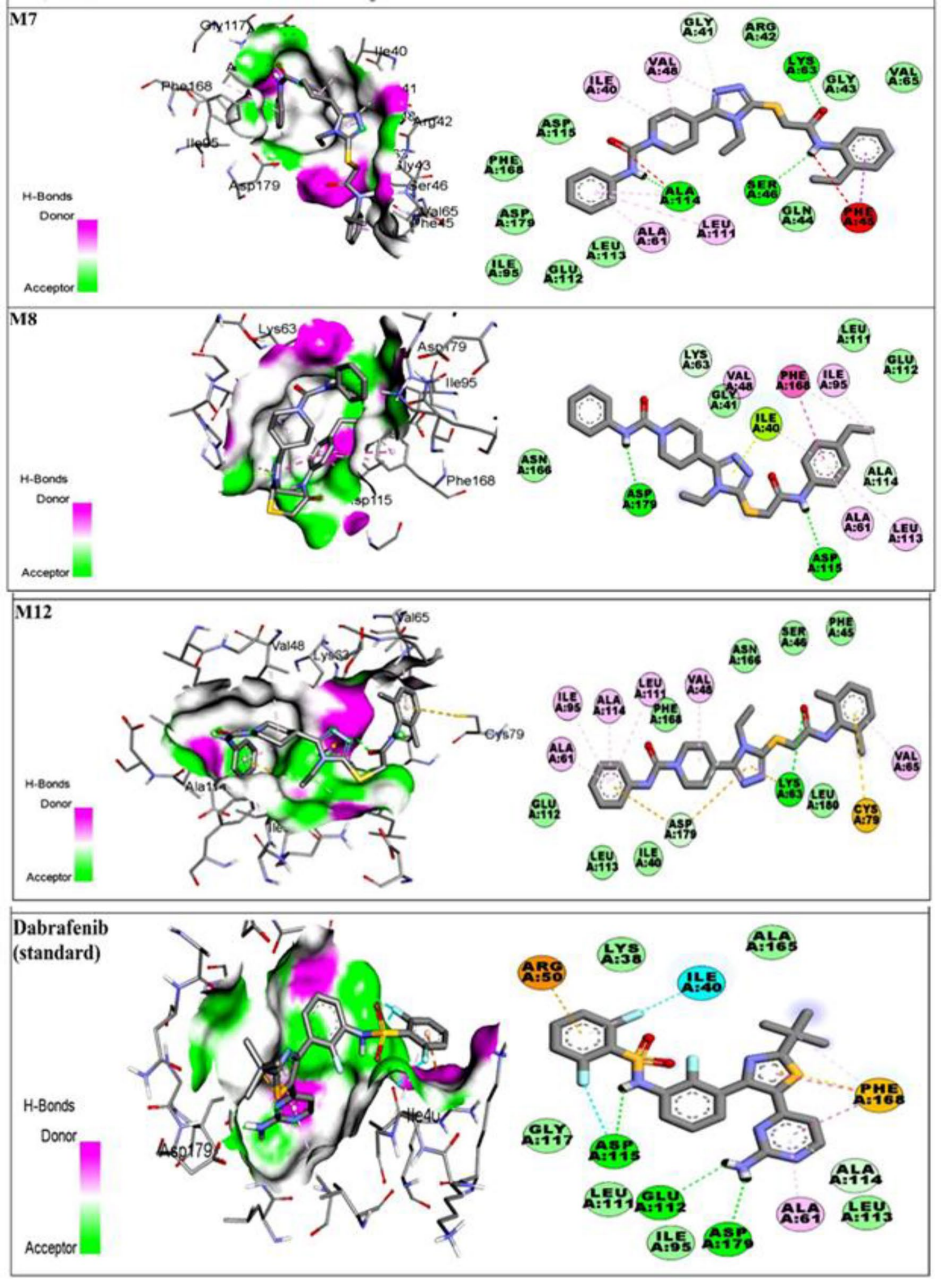
Most probable 2D and 3D interactions of compound M7, M8, M12 and Dabrafenib.

In order to determine the inhibition potential of compound with reference to standard FDA approved inhibitor, we have docked Dabrafenib within activation loop of NEK7. Docking score from both software’s was obtained as − 33.22 and − 33.51 kJ/mol, respectively. The 2D and 3D interactions were visualized using discovery studio visualizer. Docked conformation of Dabrafenib showed potent hydrophobic and hydrogen boding interaction with primary amino acid residues of active site. Hydrophobic interactions were included π-cation interaction with ARG50, halogen interaction with ILE40, one π-alkyl, two π–π T shaped and one π-sulfur interaction with PHE168. Moreover, aromatic rings of Dabrafenib were involved in π-donor hydrogen bonding with ALA114. In addition to these interactions, standard was producing three conventional hydrogen bonds with ASP115, GLU112 and ASP179. Other amino acids involved in 6 van der Waals interactions were LEU113, ILE95, LEU111, GLY117, ALA165 and LYS38. Most probable 2D and 3D interactions of compound M7, M8, M12 and standard Dabrafenib is shown in Fig. 7.

### SeeSAR analysis

**Figure 8:**
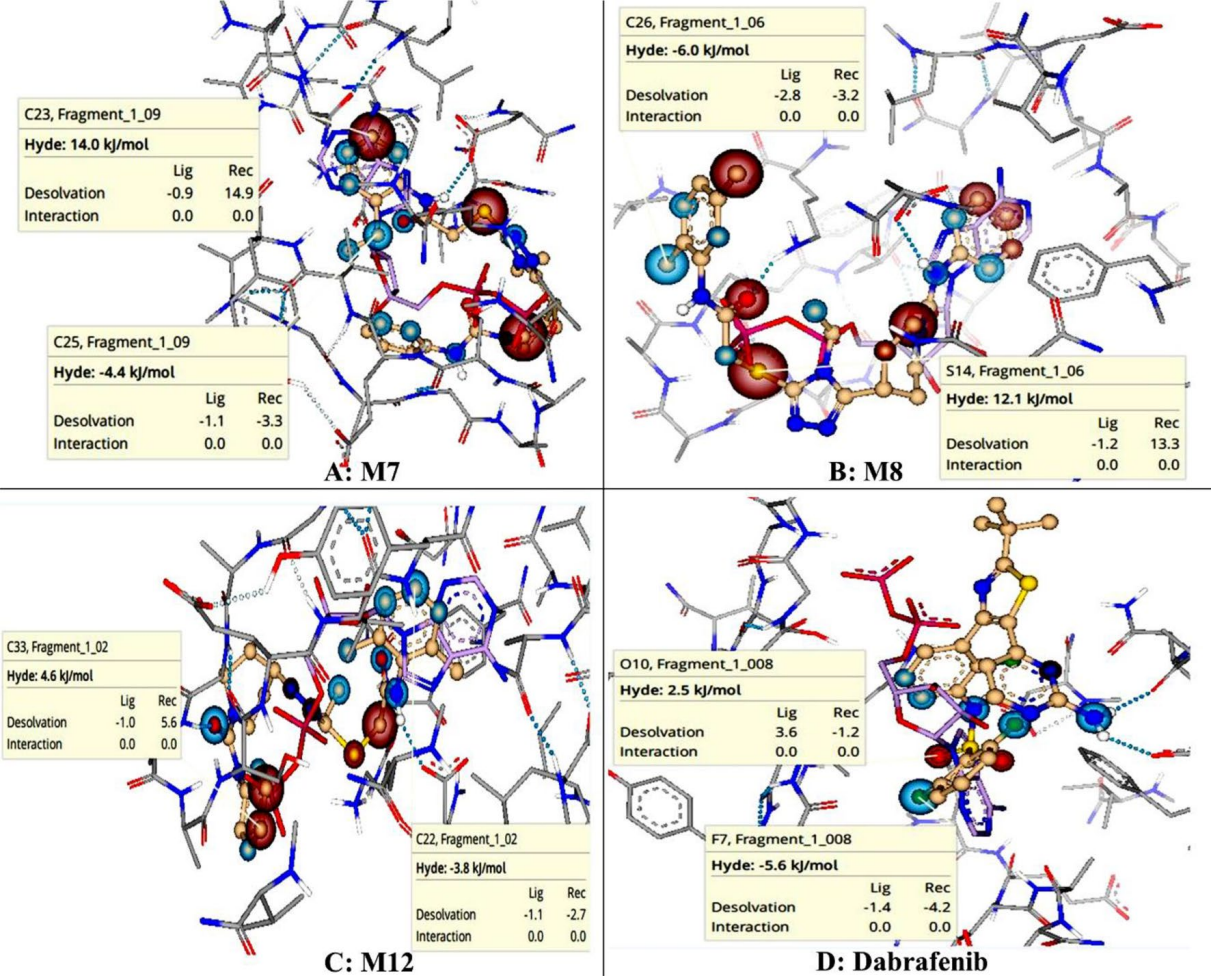
SeeSAR analysis of potent derivatives and standard; (**A**) M7, (**B**) M8, (**C**) M12, D) Dabrafenib, red colored coronas are indicating unfavorable features whereas blue colored coronas are indicating favorable contributions and colorless coronas are showing no contribution of structural components.

SeeSAR analysis of most potent derivatives was carried out which provided virtual display of binding affinities. The structural components of compounds which were contributing favorably were indicated as blue colored coronas whereas those components having negative impact on binding affinities were shown as red colored coronas. Structural components having no contribution were colored as colorless coronas. Size of corona is predictive of contribution of structural component^54^. SeeSAR visualizations of potent derivatives (Fig. 8)**,** which shows that approximately all structural features were contributing favorably but only thiol group of 1,2,4 triazolyl ring and piperdinyl ring was contributing negatively (indicated by red coronas) due to high desolvation energy. Approximately all structural features of Dabrafenib show positive contribution (blue coronas). In addition, Hyde energies of favorable coronas (blue colored) for compound M7, M8 and M12 were comparable to standard Dabrafenib i.e., − 23.43 kJ/mol.

### Molecular dynamic simulation

#### Analysis of RMSD and RMSF

**Figure 9:**
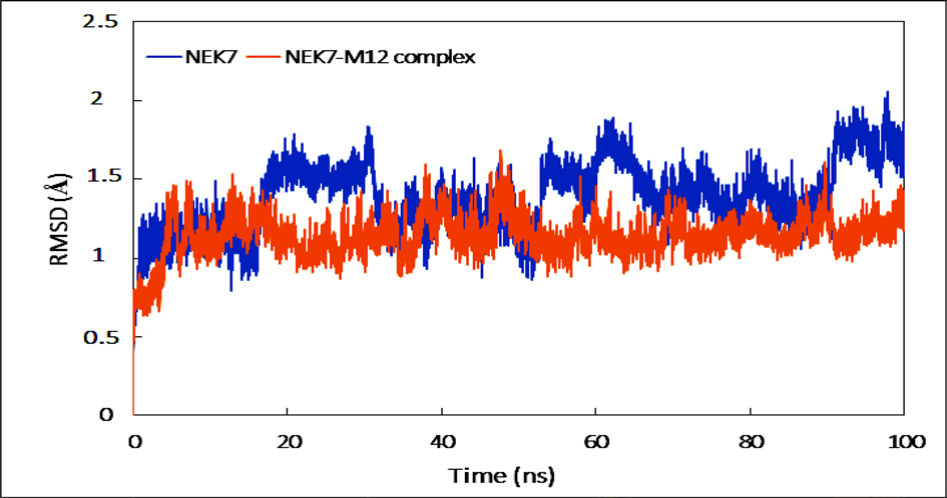
Root mean square deviation (RMSD) of backbone of NEK7 and NEK7-M12 complex as function of time.

Calculating the RMSD of each system's backbone served as a preliminary study of the trajectories. Figure 9 shows the RMSD of NEK7 and the NEK7-M12 complex as a function of time. Throughout the simulation period, both systems showed small variances but stayed rather steady. During MD modelling, the RMSD of both systems stayed less than 2 Å, showing that they are highly stable under aqueous environments^55^. The average RMSDs of the NEK7 and NEK7-M12 complexes were found to be 1.27 Å and 1.22 Å, respectively.

**Figure 10:**
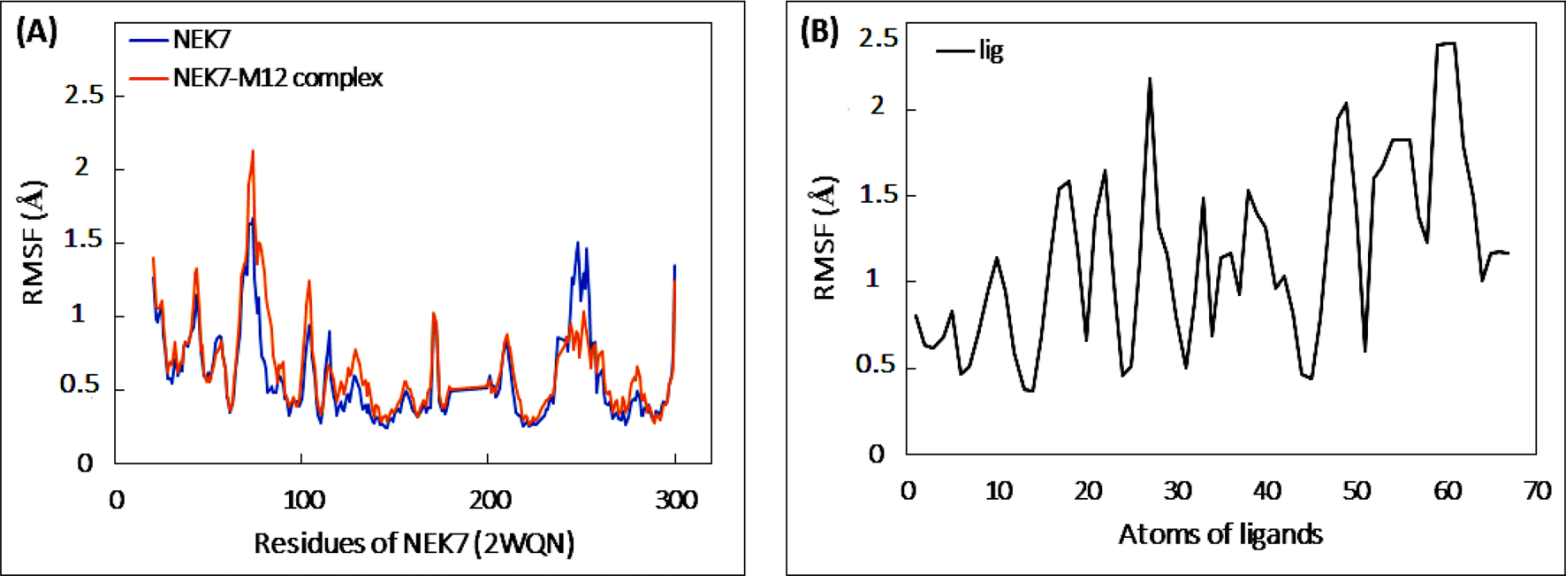
(**A**) Root mean square fluctuation (RMSF) of Cα atoms of NEK7 in the absence and presence of M12. (**B**) RMSF of each atom of M12.

Calculating the RMSF provided more insight into the MD simulation results. Figure 10A shows the RMSF of Cα atoms in each NEK7 residue in the absence and presence of M12. The RMSF of virtually all NEK7 residues was less than 2 Å, indicating that both systems are generally stable^56^. Some residues in NEK7 alone and in the NEK7-M12 complex showed higher\volatility, which might be owing to their terminal location or because they are part of random coils or turns that fluctuate. The RMSF of individual atoms of M12 was also calculated to analyze its dynamics and the result is presented in Fig. 10B. Majority atoms of the ligand fluctuate which indicate the dynamical shift from its initial position^57^. Moreover, the fluctuation in atoms of the ligand shows the movement of ligand within the binding site which was confirmed by visualizing the trajectory in VMD.

### ADMET properties

Determination of biochemical process from drug administration to elimination plays important role in lead optimization. Most of the compounds had excellent pharmacological activity but cannot be accepted due to their poor absorption, distribution, metabolism, excretion, and toxicity issues, which are known as ADMET properties. An ideal drug candidate should be administered and absorbed properly into systemic circulation and must be non-toxic and eliminate without affecting the biological activity. These processes are seems to be distinct but they are closely interrelated, so determination of ADMET properties have prime importance in drug discovery process. Since traditional methods were time consuming and many researches till 1990 went into vain due to appearance of undesirable effects in the middle of drug discovery process. So, physicochemical properties, absorption, distribution, metabolism, toxicity, excretion and medicinal properties of 1,2,4 triazole acetamide derivatives were calculated through online web server ADMETlab 2.0. It is an integrated online platform for accurate and comprehensive prediction of ADMET properties. ADME factors that were considered were physicochemical properties, blood brain barrier(BBB), Caco-2 permeability, volume of distribution(VD), P-glycoprotein (PGP) substrate, P-glycoprotein (PGP) substrate, plasma protein binding, Human intestinal absorption (HIA), MDCK permeability, Clearance (CL), Half-life (T1/2), eye corrosion, eye irritation, respiratory toxicity, AMES toxicity, carcinogenicity and synthetic accessibility score. Total 15 derivatives were subjected ADMET study using online web server ADMETlab 2.0. It was observed that all compounds had a positive computed value of human intestinal absorption (HIA), indicating that they can penetrate the intestinal membrane more easily. Furthermore, compound M7 and M12 had a greater value for HIV than standard Dabrafenib. A substance with a positive blood brain barrier value has a higher lipophilicity profile and can easily absorb from plasma membranes. The calculated value for the blood brain barrier (BBB) and blood placental barriers (BPB) were shown to have a high likelihood of being BBB positive. In terms of plasma glycoprotein (PGP) substrate and inhibitor, it was discovered that the output value of all compounds had probability of being a PGP substrate or inhibitor specifically compound M7, M8 and M12. PPB (plasma protein binding) is a significant component in determining drug safety, since compounds with a high PPB value (> 90%) have a narrow therapeutic index, whereas treatments with a low PPB value are considerably safer. Only compound M2 had low PPB values in this investigation, indicating that these drugs have a broad therapeutic index. PPB values of more than 93 percent were found in all compounds, indicating that they have a narrow therapeutic index. Comprehensive ADMET properties are given in “Supplementary Data S1”. It was observed that physicochemical properties of all compounds were meeting the criteria of drug like rule i.e., Lipinski rule of five as listed in Table 4.

**Table 4:** Physicochemical properties of compounds.

Physicochemical properties								
	Molecular weight	Density	nHA	nHD	TPSA	LogS	LogP	LogD
M1	444.23	0.994	8	1	83.36	− 2.714	2.431	2.201
M2	430.22	1.001	8	2	92.15	− 2.764	2.218	2.477
M3	470.25	0.994	8	2	92.15	− 4.044	3.296	3.156
M4	478.22	0.991	8	2	92.15	− 3.877	2.789	2.822
M5	478.22	0.991	8	2	92.15	− 4.075	3.155	2.832
M6	478.22	0.991	8	2	92.15	− 4.813	3.649	3.228
M7	492.23	0.985	8	2	92.15	− 4.636	3.751	3.236
M8	492.23	0.985	8	2	92.15	− 5.34	4.135	3.433
M9	492.23	0.985	8	2	92.15	− 4.625	3.797	3.089
M10	492.23	0.985	8	2	92.15	− 4.431	3.706	3.084
M11	492.23	0.985	8	2	92.15	− 4.527	3.782	3.152
M12	492.23	0.985	8	2	92.15	− 3.849	3.076	2.958
M13	492.23	0.985	8	2	92.15	− 5.231	4.251	3.447
M14	464.2	0.998	8	2	92.15	− 4.475	3.063	2.937
M15	492.23	0.985	8	2	92.15	− 4.527	3.782	3.152

### Cell viability assay

**Figure 11:**
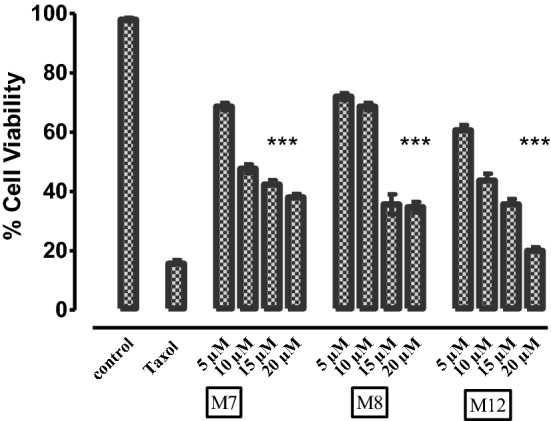
% Cell viability of HepG2 cells after treatment with potent derivatives (M7, M8 and M12) by using Taxol as Positive Control. The cells were treated with four different concentrations (5 µM, 10 µM, 15 µM and 20 µM) for 48 h and cell viability was measured by MTT assay. Data were analyzed as mean of three experiments ± S.D. (n = 3) by using PRISM 5 (GraphPad, San Diego, California, USA).

To support the in-silico studies, the preliminary screening of the most potent derivatives was carried out using in-vitro cell viability assay (MTT assay). The Human HepG2 liver cancer cells were treated with four different concentrations of the selected compounds i.e., 5 µM, 10 µM, 15 µM and 20 µM. The concentrations were selected based on the predicted inhibitory values obtained during docking studies. A linear response of cell death was observed by each derivative. Derivative M12 showed maximum cell death justifying the computational studies where this compound was found best inhibitor of NEK7. The single concentration taxol was used as positive control. The results were calculated by comparing with the total activity control (without inhibitor) i.e., un-treated cells. The % viability graph was generated via graph pad Prism software and is given in the Fig. 11.

## Conclusion

The current work used a thorough in-silico strategy to find strong and selective NEK7 protein inhibitors. The chemical reactivity and stability of the compounds were measured using density functional theory studies. In addition, FMOs and MEP were computed, confirming the chemical reactivity of the compounds. Two docking software’s, MOE and Autodock, were used to conduct molecular docking investigations in order to improve the accuracy of in-silico molecular modelling. The majority of compounds produced stable protein–ligand complexes with high binding energies, specifically compound M7, M8, and M12 were forming the most stable complexes with the highest binding energies. The most stable compound with the highest binding energy was then subjected to MD simulation experiments, which provided information on the complex's stability over time. MD simulations demonstrated that the complex was in stable state. To establish the physicochemical attributes and toxicity profile of compounds, thorough ADMET studies were conducted, which investigated the compounds' toxicity, drug similarity, and synthetic accessibility. At the end, the results were supported by cell viability assay where the M12 was identified as the best inhibitor of NEK7. Conclusively, all the compounds had a low toxicity profile and acceptable synthetic accessibility score. NEK7 is a potential and selective target for 1,2,4 trizole acetamide derivatives, according to findings of current study.

## Materials and methods

### Phenylcarbamoylpiperidine-1,2,4-triazole amide derivatives

A general procedure for the synthesis of phenylcarbamoylpiperidine-1,2,4-triazole derivatives has been reported in our previous work^39^. The goal of this research is to look at the *in-silico* inhibitory potential of novel amide analogues of phenylcarbamoylpiperidine-1,2,4-triazole with diverse substituents (M1–M15). Phenylcarbamoylpiperidine-1,2,4-triazole amide derivatives along with their previous anti-inflammatory activity against 15-LOX are listed in Table 5.

**Table 5:** Chemical formula and 15-LOX inhibitory profile of all derivatives.

Compound	Chemical formula	15-LOX inhibitory profiles (IC_50_ µM)
M1	C22H32N6O2S	45.62
M2	C21H30N6O2S	17.5
M3	C24H34N6O2S	36.24
M4	C25H30N6O2S	45.67
M5	C25H30N6O2S	35.61
M6	C25H30N6O2S	97.64
M7	C26H32N6O2S	36.52
M8	C26H32N6O2S	42.95
M9	C26H32N6O2S	Inactive
M10	C26H32N6O2S	97.62
M11	C26H32N6O2S	Inactive
M12	C26H32N6O2S	98.63
M13	C26H32N6O2S	105.43
M14	C26H32N6O2S	89.56
M15	C24H28N6O2S	108.73
Dabrafenib	C23H20F3N5O2S2	–
Quercetin	C15H10O7	4.86

### Computational studies

Comprehensive in-silico investigations were performed with the NEK7 protein to examine the anti-cancer potential of 1,2,4 triazole acetamide derivatives. Molecular docking research, extensive density functional theory investigations, including global and local reactivity descriptors, chemical softness, chemical hardness, and electrostatic potential and Molecular dynamic simulations were among the computational studies. ADMET characteristics were computed using ADMETlab 2.0 to establish the ADME profile of all derivatives^58^.

### Density function theory (DFT)

The density functional theory was used to optimize the gas phase geometries of the investigated compounds in both gas and solvent phases (DFT). The molecular geometry parameters, frontier molecular orbital (FMO), global and local reactivity descriptors, and molecular electrostatic potential were all collected using DFT (MEP). All of these computations were done with the Gaussian09^59^ software, which used the Becke-3-Parameter-Lee–Yang–Parr^50^ (B3LYP) method. For all calculations, the SVP basis sets were employed. Gauss View 6 was used to visualize the output files^60^.

### Molecular modeling

#### Preparation of protein

The protein data bank (www.rcsb.org, PDB: 2WQN, resolution: 2.30) was used to derive the crystal structure of human NEK7 protein. The downloaded protein was generated independently in both docking softwares, Autodock^48^ and MOE^49^. The Autodock suite was used to prepare the protein, which involved removing water molecules, adding polar hydrogen atoms, and assigning partial charges to each atom. Autodock tools were also used to prepare missing residues. During the protein production procedure, complexed adenosine diphosphate (ADP) and het atoms were removed from NEK7. Following protein preparation, previously synthesized 1,2,4 triazole acetamide derivatives were selected as testing ligands and docked with NEK7. The active site was selected using rectangular coordinates based on the co-crystal ligand ADP. Grid box XYZ dimensions were set to − 12.348, − 33.512, and − 48.605, respectively. The search spacing was adjusted at 0.55 Å in order to cover the largest number of amino acids in the activation loop. The number of points in the X, Y, and Z dimensions, on the other hand, were set to 60, 60, and 60, respectively. The number of docking postures was set to 100 with a population size of 300 to cover all potential ligand binding locations. Following the establishment of the necessary parameters, ligands were docked with protein in order to identify probable hits and generate reliable binding poses. The binding poses of the compound with the highest docking score were visualized using Discovery Studio Visualizer 17.2.

MOE's protein preparation module was used to inspect the protein structure for any missing atoms or residues and make any necessary adjustments. MOE's protein preparation process comprised applying gas tier charges through the MMFF94x forcefield, adding hydrogen atoms, removing water molecules, 3D protonation of the structure, and minimizing the protein structure to a chosen gradient. The binding site of NEK7 was defined using the original co-crystal ligand (ADP). The dummy atoms were generated at the binding location using MOE's site finder program. The docking algorithm was configured to use the triangle matcher placement approach^61^. Furthermore, the induced fit refinement approach was applied to generate docking positions. The triangular match algorithm was programmed to create 100 poses, but the induced fit refinement approach produced ten poses. Furthermore, the default GBVI/WSA dG technique was used as a docking function in MOE^62^. Autodock and MOE docking conformations with the lowest binding energies were chosen for further study.

#### Preparation of ligands

Ligand databases were created by generating the structures of N-alkyl/aralkyl/aryl acetamide triazole derivatives from Saima, Muzaffar et al.^39^. These molecules were previously tested for their ability to inhibit the 15-lipoxygenase enzyme. Chemdraw Ultra 12.0^63^ was used to draw the structures of all derivatives based on the IUPAC name of the compounds. The aryl ring was replaced with an ethyl group, and 3D optimization and energy reduction were performed at 0.1 gradient using Chem3D pro 12.0^64^. Compounds were saved to SDF format after energy reduction. These SDF files were then transformed into the appropriate format for the docking programme, such as PDBQT for Autodock and SMILES for SeeSAR analysis. These modifications were carried out with the help of the OpenBabel GUI^65^.

### Molecular docking protocol

The Autodock and MOE docking modules were used to perform docking-based virtual screening on the targeted NEK7 protein. Compounds were docked into a protein's active pocket. For visualization and posture creation, the compound with the highest binding energy was chosen.

### Visualization

Autodock docking conformations were displayed using Discovery studio visualizer 17.2^66^. The best docked conformation's 2D and 3D interactions were generated using the Discovery Studio visualizer. MOE, on the other hand, has an inbuilt visualizer tool that may be used to visualise 2D and 3D interactions of the best docked conformation.

Furthermore, to identify a persuasive rationale for NEK7 inhibitor binding affinity, SeeSAR analysis of the most active and least active ligands was performed using SeeSAR software^67^, which gives a visual depiction of binding affinity. The structural features of ligands that were not contributing favourably to overall binding affinity were indicated by red coloured coronas, whereas structural features of ligands that were contributing favourably were indicated by blue coloured coronas; the larger the corona, the greater the contribution. No-contribution structural elements were not coloured. SeeSAR was used to create a total of ten docking postures for each ligand^54^.

### Validation

Validation of docking protocol was done by redocking co-crystal ligand and Dabrafenib into the active pocket of NEK7 protein. Assessment was done on the basis of calculated RMSD value. Only those docking poses were considered successful whose RMSD value of docking pose and the experimentally determined conformation of a ligand was less than 2.0 Å^68^.

### Molecular dynamic simulation

Molecular dynamics (MD) simulations were used to investigate the stability and interaction of the hit molecule (M12) acquired from prior research. Gromacs-2018.1 was used to run the MD simulations, which used the amber99sb-ILDN force field^69,70^. For simulation investigations, the docked complex was used as the starting point. The topology of M12 was created using AmberTools21's Antechamber and the AM1-BCC charge model^71^. TIP3P water model was used to solvate NEK7 and its complex with M12, and then the energy of each system was reduced using steepest descent minimization to eliminate the weak Van der Waals connections. Both systems were then equilibrated in two processes. First, NVT was equilibrated at constant temperature and volume for 1 ns using a V-rescale thermostat^72^. The Parrinello-Rahman barostat was used to produce the second equilibration for NPT at constant pressure and temperature for 1 ns^73^. The equilibration coordinates were used to run a 100 ns MD simulation, with 10,000 frames of each system stored from their individual trajectories. Before analysis, both trajectories were treated to PBC adjustments. Gromacs tools were used to conduct all of the analyses.

### ADMET properties

The determination of ADMET characteristics is critical for rolling out unfavorable effects of a drug candidate at the early stages of the drug development process. For the in-silico estimation of ADMET characteristics, efficient and accurate online prediction models were constructed. The online in-silico prediction model ADMET lab 2.0^58^ was used to determine ADMET attributes such as absorption, distribution, metabolism, excretion, toxicity, and physicochemical properties of all chemicals, and drug similarity properties were assessed using the Lipinski rule of five^58^. Chemdraw Ultra was used to convert all compounds to SMILES format, and these SMILES structures were then uploaded to the ADMET lab 2.0 web server. It also makes using the JMSE editor to create desired structures easier. Within loading the SMI structure, the submit button was used to submit the data, and it returned ADMET attributes in pdf and spreadsheet format, which could be downloaded after a few minutes.

### Cell viability measurement (MTT assay)

In order to determine the anticancer effect of the most potent compounds, the Human HepG2 liver cancer cells were cultivated in Dulbecco's Modified Eagle's Medium (DMEM) supplemented with 10% fetal bovine serum (FBS), 100 units/ml penicillin, and 100 g/ml streptomycin and kept at 37 °C in a humidified environment with 5% CO_2_. Cells were treated with compounds dissolved in DMSO with the final concentration of DMSO (0.05%). The MTT assay was performed as discussed earlier^74^. In a brief, HepG2 cells were treated for 48 h with various doses of the potent derivatives. Then 10 µl solution of MTT (5 mg/mL) was added to each well after 48 h of post-incubation. The plate was covered with aluminium foil and kept in the incubator at 37 °C for about 4 h. The medium was taken out and replaced with 150 µL of DMSO to dissolve the remaining purple formazan precipitate that was still there. A microplate reader (Thermo Scientific) was used to record the absorbance at 570 nm. This colorimetric assay looked at how the mitochondrial enzymes of the metabolically active cancer cells reduced MTT to purple formazan, which made the cancer cells look more alive. The Cell viability was determined as a percentage. The experiment was done three times, and the results are shown in standard deviation and mean values.

## Supplementary Information

Supplementary Information.
