# Supplementary material for: Identification of potent inhibitors of NEK7 protein using a comprehensive computational approach
Source: Sci Rep. 2022 Apr 18;12:6404. doi: 10.1038/s41598-022-10253-5 (PMC9016071; doi:10.1038/s41598-022-10253-5)
Supplement: Supplementary file 1 — Supplementary Information. [file 41598_2022_10253_MOESM1_ESM.docx]

**Identification of potent inhibitors of NEK7 protein using a comprehensive computational approach**

**Mubashir Aziz^1^, Syeda Abida Ejaz^1*^, Nissren Tamam^2^, Farhan Siddique^3,4^, Naheed Riaz^5,^ Faizan Abul Qais^6^, Samir Chtita^7^, Jamshed Iqbal^8^***

*^1^ Department of Pharmaceutical Chemistry, Faculty of Pharmacy, The Islamia University of Bahawalpur, Bahawalpur 63100, Pakistan*

*^2^ Department of physics, College of Science, Princess Nourah bint Abdulrahman University, P.O*

*Box 84428,Riyadh 11671,Saudi Arabia*

*^3^Laboratory of Organic Electronics, Department of Science and Technology, Linköping University, SE-60174 Norrköping, Sweden*

*^4^ Department of Pharmacy, Royal Institute of Medical Sciences (RIMS) Multan 60000, Pakistan*

*^5^Department of Chemistry, Baghdad-ul-Jadeed Campus, The Islamia University of Bahawalpur, Bahawalpur 63100, Pakistan*

*^6^Department of Agricultural Microbiology, Faculty of Agricultural Sciences, Aligarh Muslim University, Aligarh, UP 202002, India*

*^7^ Laboratory of Physical Chemistry of Materials, Faculty of Sciences Ben M’Sik, Hassan II University of Casablanca, BP7955 Sidi Othmane, Casablanca, Morocco.*

*^8^Centre for Advanced Drug Research, COMSATS University Islamabad, Abbottabad Campus, Abbotabad, Pakistan*

**Corresponding Authors**:

Syeda Abida Ejaz; [abida.ejaz@iub.edu.pk](mailto:abida.ejaz@iub.edu.pk); [abidaejaz2010@gmail.com](mailto:abidaejaz2010@gmail.com)

Jamshed Iqbal; [jamshediqb@gmail.com](mailto:jamshediqb@gmail.com)

**Supplementary Data**

**Graphical Abstract**

**
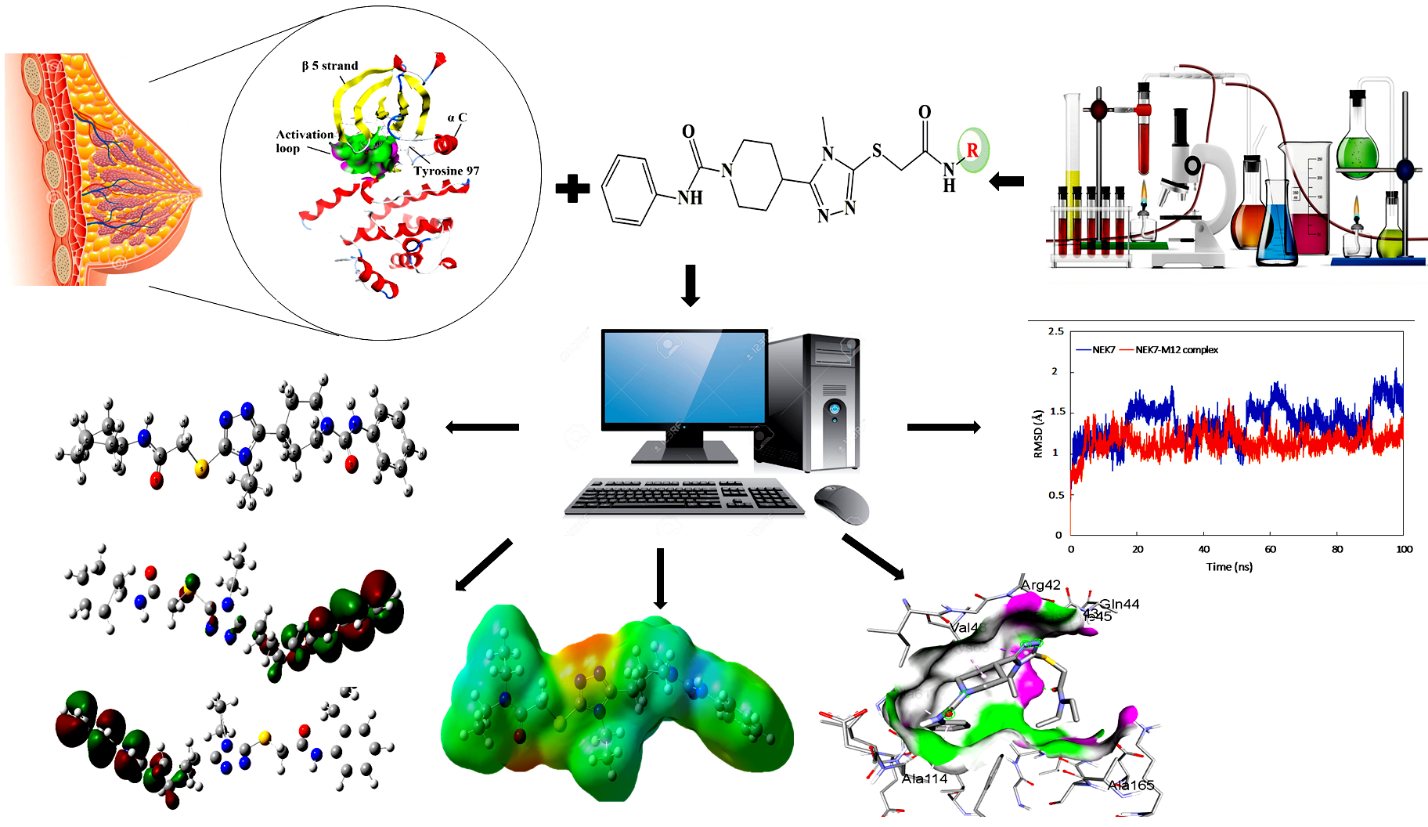
**

In-silico approach in investigation and evaluation of Phenylcarbamoylpiperidine-1,2,4-triazole amide derivatives (M1-15) as potent anti-cancerous agent

**Introduction**

Tyrosine 97 residue, which points into the active site and distorts intact DFG/DLG motifs, naturally renders an inactive shape of NEK7 protein. This is an autoinhibitory mechanism that regulates NEK7 protein expression during the cell cycle. In 2009, efforts were attempted to crystallise the NEK7 adenosine diphosphate (ADP) bounded structure, which could serve as a structure-guided strategy for developing effective inhibitors. Given the lack of a ligand-bounded structure for NEK7, Byrne, Matthew J., et al^1^ attempted to identify novel beginning sites for NEK7 inhibitor binding. They were successful in crystallizing the NEK7 protein using compound 51 (Figure S1). The NEK7 protein's ligand-bounded structure was the first to be discovered^1^. These discoveries aided us in identifying the NEK7 activation loop, where we may conduct in-silico investigations using standard dabrafenib and polyfunctional heterocyclic drugs. Furthermore, these findings offered a compelling foundation for the current study's approach. The inhibitors discovered in this work have the potential to serve as a lead for the creation of effective anticancer medicines.

**Figure S1.** Structures of known inhibitors of NEK7 ^1^

To begin, an exhaustive examination of the literature review was done to gain a better understanding of the NEK7 protein's activation and inhibition mechanisms. The NEK protein's catalytic site is composed of two lobes connected by a flexible hinge. These three structures combine to produce the NEK protein's active pocket, which is where ATP binds and phosphorylation occurs^2^. The activation of a kinase may be mediated by ATP phosphorylation or by protein-protein interaction. Activated NEK7 protein has complete HRD and DFG motifs, as well as a well-formed R spine^1^. Following the identification of the active site, we conducted a structure-based virtual screening.

**Results and Discussion**

Prior to virtual screening, a computer-aided molecular modelling strategy should include comprehensive research on the active pocket and structure of the targeted protein^3^. As a result, the crystallographic structure of the NEK7 protein was studied. The human NEK7 (h-NEK7) protein is a 34.5kDa protein with a single canonical kinase domain. It is a tiny protein with a single catalytic domain in the N-terminal lobe of 30-40 amino acids^1^. The N-terminal lobe of the hNEK7 protein was made up of short 5 strands that were coupled to the bigger C-terminal lobe. The C lobe was dominated by the C helix. The ATP binding pocket formed by the intermediate cleft between the C and N terminal lobes facilitates the transfer of phosphoryl groups from ATP to protein, resulting in active conformation^4^. Because N terminal extensions are not conserved, it has been hypothesized that they play a crucial role in differential kinase regulation^2^. The N terminal activation loop contains DFG/DLG motifs, which are triads of essential amino acids. Aspartic acid, Phenylalanine, and Glycine were found in DFG motifs, whereas Aspartic acid, Leucine, and Glycine were found in DLG motifs. Within NEK7's activation loop, these essential amino acids formed a catalytic trio. A lysine in the DFG motif, for example, maintains the phosphates of ATP in place, while Glutamic acid on the alpha C helix of the N lobe played a part in keeping that connection in place. Furthermore, a divalent cation connected with the g-phosphate of the ATP molecule was induced by an aspartic acid of DLG conserved patterns. To generate a platform for substrate binding, DFG/DLG motifs must be phosphorylated. Furthermore, NEK7 protein research indicated that it has two hydrophobic R and C spines that cross the protein's C and N terminal lobes. Within the catalytic domain, these hydrophobic spines include four residues that must be appropriately stacked in order to maintain active conformation (Figure S2). The activation loop might be phosphorylated by an ATP molecule to achieve proper stacking of residues^3^. Two hydrophobic spine residues were found in the C lobe and two more in the N terminal lobe of the protein. Residue 1 (RS1) was the initial HRD motif residue, while Residue 2 (RS2) was the centre DFG motif residue, Residue 3 (RS3) was found in the alpha C helix (LEU86), and Residue 4 (RS4) was found at the end of the N terminal in 5 strands^5^.


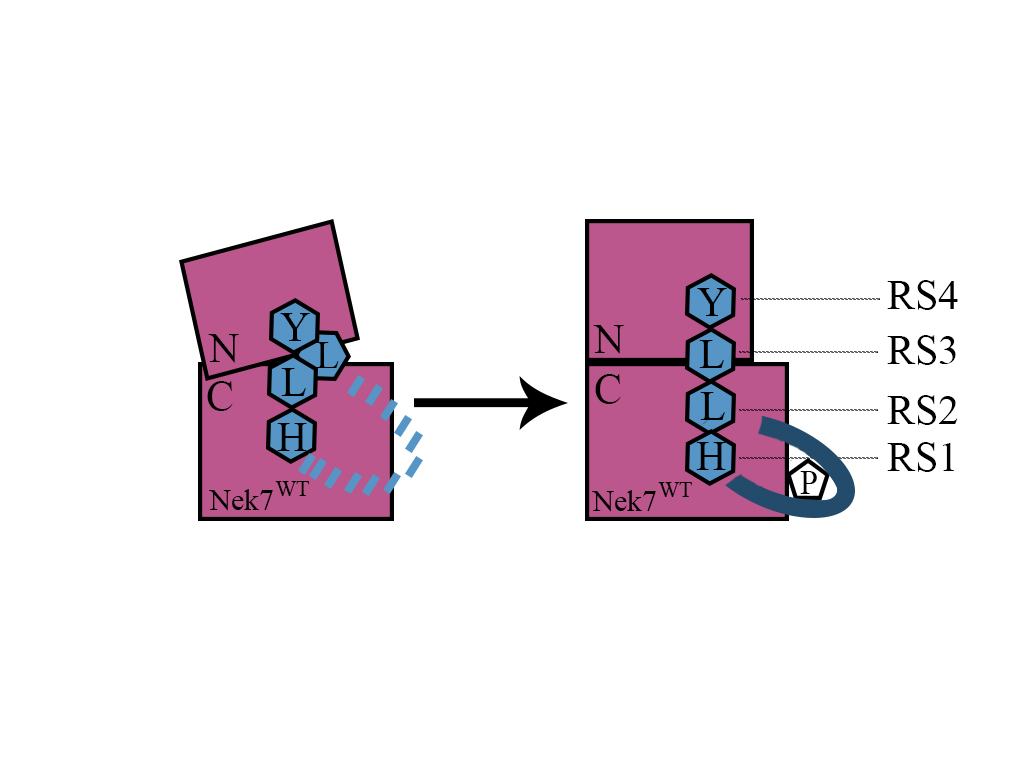


**Figure S2**: Proper stacking of hydrophobic spine residues of NEK7 which was induced by Phosphorylation. Proper stacking is necessary for active conformation of protein.

Furthermore, a tyrosine residue (TYR97) was discovered in 10% of human kinases as an auto inhibitory mechanism that kept NEK7 in a catalytically inactive state. Tyrosine residues point into the protein's activation loop, forming hydrogen bonds with DLG/DFG motifs and limiting the C helix's inward active conformation. The DFG/DLG motifs have been broken by the enzyme's inactive conformation. The DFG/DLG inside the activation loop of the NEK7 protein was properly oriented in the active conformation. Furthermore, the active conformation of the NEK7 protein showed inward migration of the alpha C helix and the creation of salt bridges between DFG/DLG motif amino acids. (Figure S3).

**
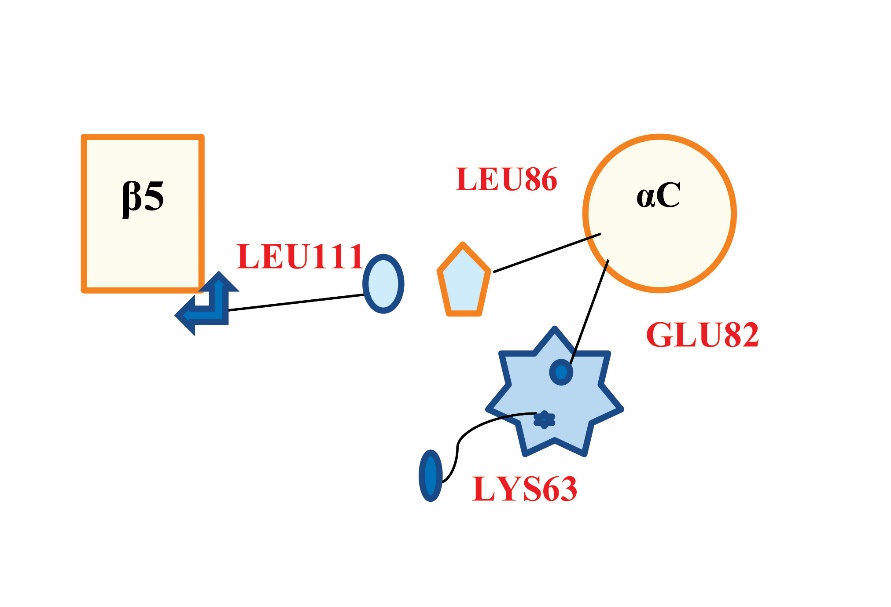
**

**Figure S3**: Schematic representation of active conformation of NEK7 protein, β 5 strand is shown as rectangle shape whereas alpha C helix is shown as a circle. Equivalent residues are represented by lines, formation of salt bridge between GLU82 and LYS63 is shown by transparent blue star whereas closely packed gatekeepers LEU111 and LEU86 are shown as circle and a ring.

Residues of amino acids within the activation loop, LYS63 and GLU82 formed an active salt bridge. The phosphorylation of DFG/DLG motifs by an ATP molecule stimulated the creation of salt bridges between two amino acids. Another notable aspect of the active conformation of the NEK7 protein was the close packing of LEU86 and LEU111, which resulted in the development of a hydrophobic core that allows salt bridges to form. Within the activation loop, these two leucine residues were identified as gate keepers (Figure S4). For catalytic activity, salt bridges and tight packing of leucine residues were essential^6^**.**


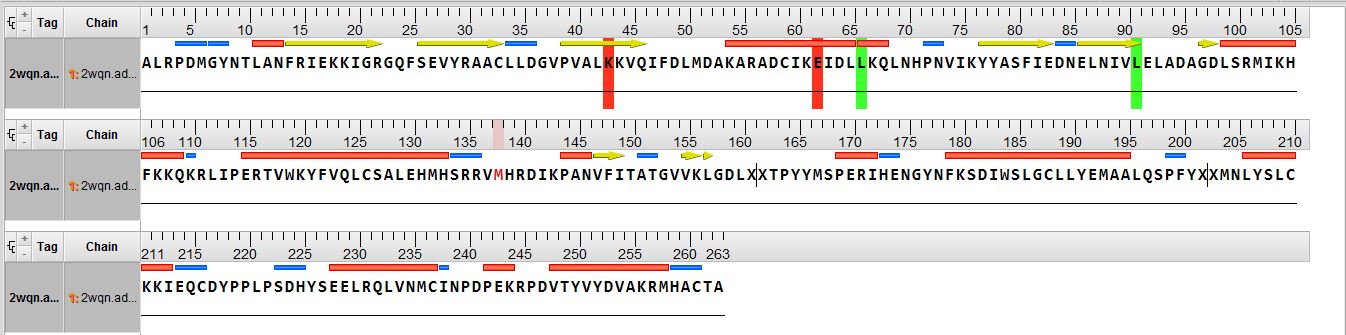


**Figure S4**. Protein sequence of NEK7: LYS63 and GLU82 are indicated as red boxes whereas LEU86 and LEU111 are shown as green boxes.

The ADP molecule is a co-crystal ligand that interacts with DFG/DLG residues in the activation loop, disrupting the protein's active conformation. ADP extends into active sites from the 5 strand and alpha Cα helix, forming hydrogen bonds with LYS63, GLY43, and GLU112. The outward displacement of the alpha C helix was produced by hydrogen bonding, which destroyed salt bridges between LYS63 and GLU82, and the activation loop was shifted from its normal position. The docking score of ADP with NEK7 was discovered to be -13.76536 kJ/mol, whereas TYR97 was sandwiched between the activation loop's gate keeper residues, LEU111 and LEU86 (Figure S5). It was discovered that the hydroxyl end of TYR97 formed a hydrogen bond with the amide group of LEU180, altering the protein's active conformation.

**
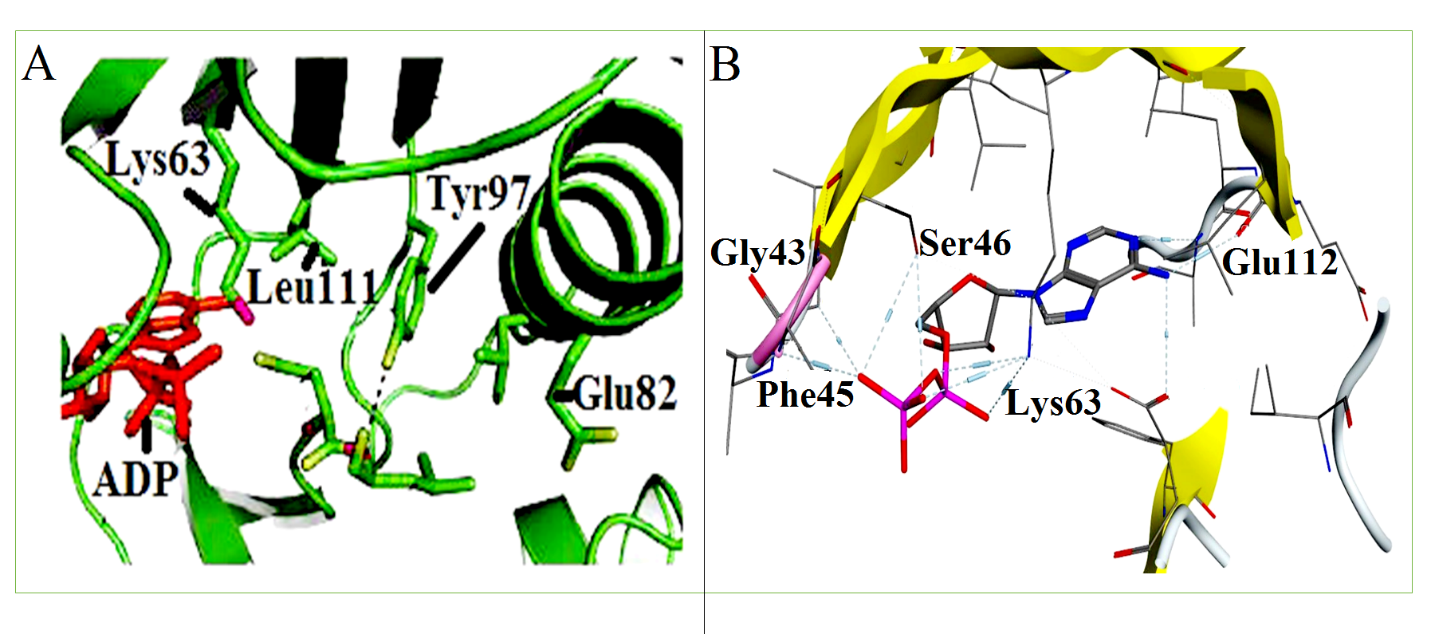
**

**Figure S5.** 3D representation of interactions. A) Interaction of TYR97 with amino acids of active site B) Interaction of ADP with amino acids of active site. Hydrogen bonding is represented by blue dotted lines.

LYS63 and GLU82, which form salt bridges during active conformation, were the most significant residues. Furthermore, during active configuration, LEU86 and LEU111 served as essential gatekeepers. LEU180, SER195, GLU112, ALA114, VAL48, SER46, ILE40, and GLN44 were also key predictors of catalytic activity in the N terminal lobe. Interactions with amino acids from DFG/DLG motifs such as LEU86, LEU111, LYS63, GLU82, VAL48, GLY43, GLU112, ALA114, SER46, PHE45, and GLN44 were clearly significant. Ligands that made bonding and non-bonding interactions with the indicated amino acids and disrupted the protein's active conformation were powerful and selective hits.

**Molecular Docking**

Compound M5 has an aromatic benzene ring substitution, as well as a methyl group substitution in the ortho position of the benzene ring. This occurred in the N-substituted acetamide portion of the molecule. The presence of a methyl group in the ortho position had a major impact on the compound's inhibitory capability. Furthermore, the methyl group has electron-donating properties, resulting in a positive mesomeric impact (+M) on the molecule. Interestingly, no amino acid was found to make hydrogen bonds with any portion of the molecule. Hydrophobic interactions made up the majority of the interactions. Alkyl, -alkyl, -cation, -stacked, and van der Waals interactions were among the hydrophobic interactions studied. The phenyl ring of the N-substituted acetamide component of the chemical and ALA61 and ALA114 of the catalytic site of the protein showed -alkyl interaction. DLG/DFG contains both amino acids. loop of activation motifs The -alkyl interaction, which involves the electronic cloud of an aromatic ring and the alkyl portion of an amino acid residue, is an essential stabilising contact. Furthermore, with ALA165 and PHE168, the core 1,2,4 triazolyl-3-thiol ring formed -alkyl and -stacked connections. Both interactions stabilised the protein-ligand complex and kept the protein in an inactive state. Another major hydrophobic contact was discovered between the compound's piperdinyl ring and the active site's ALA165. The phenyl ring of the N-phenylcarbamyl portion of the molecule was also involved in a significant -cation interaction with ARG121. A major stabilising interaction between a cation and a polarizable electronic cloud of an aromatic ring is the -cation. 11 van der waals interactions with ASN66, ASP118, GLY117, ALA116, ASP115, LEU111, LEU113, LYS63, ILE40, VAL48, and ASP179 were among the other hydrophobic contacts. LEU111 and LYS63 were complexed via van der Waal interactions; these amino acids are involved in gate keeper and salt bridge production, respectively, inside the protein activation loop. MOE and Autodock docking scores were -29.75 and -26.10 kJ/mol, respectively, which were better than co-crystal ligand ADP and equivalent to FDA-approved inhibitor Dabrafenib.

ALA61, LEU111, ASP179, PHE168, ALA114, ILE95, VAL48, GLY41, ARG42, GLY43, LYS63, SER46, PHE45, GLN44, ILE40, ASP115, and GLU112 were the amino acid residues implicated in bonding and nonbonding interactions with compound M6. M6 has an aromatic ring substitution, which is further replaced by a methyl group at the meta position. The methyl group can exert a positive mesomeric impact by donating electrons, but the location of substitution was crucial in defining the inhibitory potential of the molecule. It was clear that M6 had a lower docking energy than M5 owing to the shift in methyl group position on the benzene ring. Hydrogen bonding occurred between the carboxylate end of ARG42 and ALA114 with electropositive hydrogen atoms of N-substituted acetamide and N-phenylcarbamyl portion of M6 respectively, which was the most stabilizing contact. These amino acids, ARG42 and ALA114, are part of the activation loop's DFG/DLG motifs. Surprisingly, the phenyl ring of the N-phenylcarbamyl portion was implicated in strong hydrophobic interactions with active-site amino acid residues. It interacted with LEU111, ALA61, and ILE95 in a -alkyl interaction. Because LEU111 is a crucial gate keeper residue responsible for maintaining NEK7's active conformation, the -alkyl interaction with it is extremely important. It also had a -anion interaction with ASP179, a -stacked interaction with PHE168, and a -donor hydrogen bond with ALA114. All of these interactions are electrostatic interactions between ligand and protein that are responsible for the protein's inactive state. In addition, the carbon hydrogen bonding with GLY41 was mediated via the 1,2,4 triazolyl-3-tiol ring. 8 van der Waal interactions with GLN44, GLY43, LYS63, SER46, PHE45, GLU112, ILE40, and ASP115 were also included as hydrophobic interactions. M6 had a docking score of -31.08 and -25.81 kJ/mol from MOE and Auto dock, which was better than the co-crystal ligand ADP (figure S6).


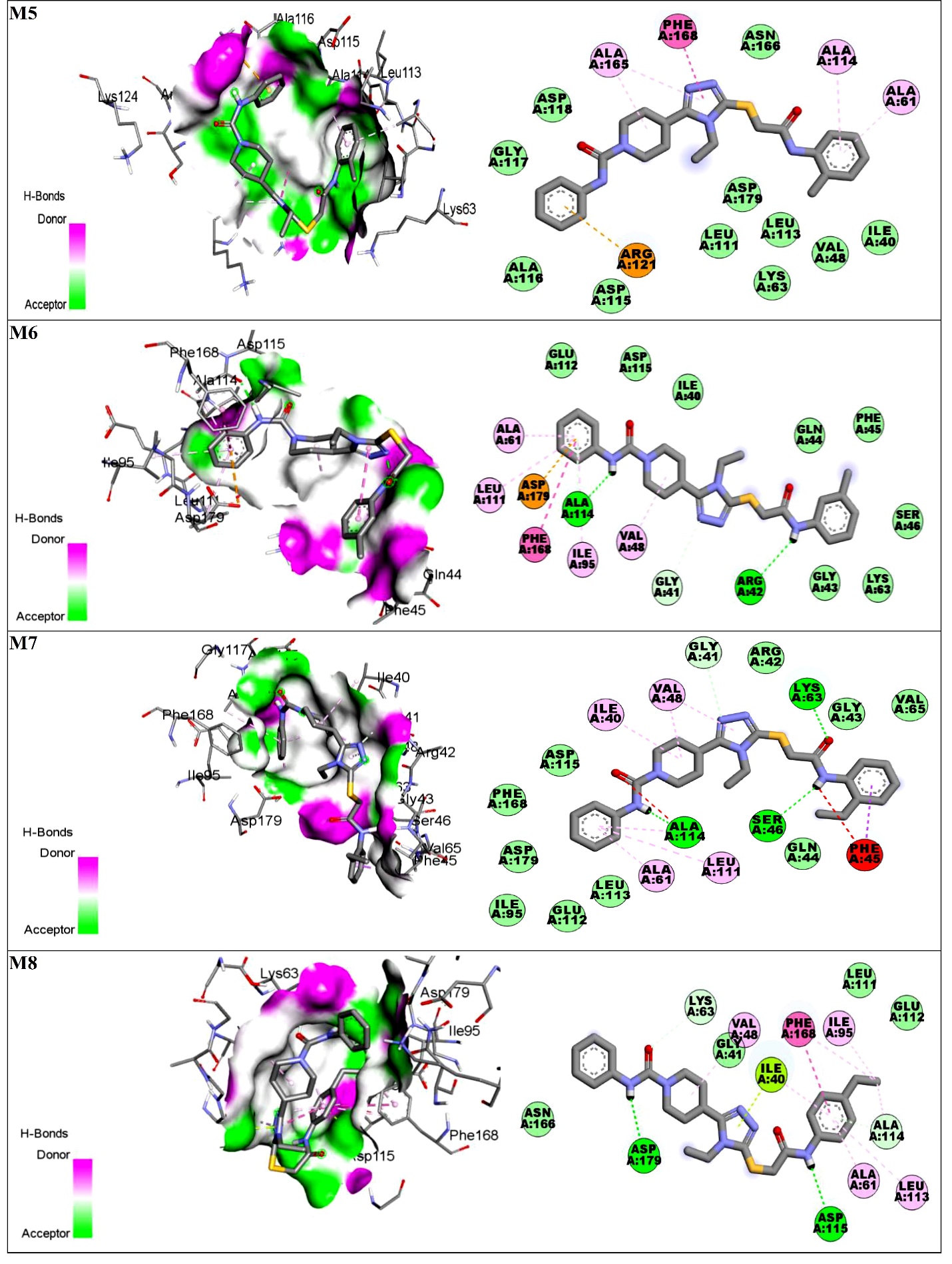


**Figure S6.** Most probable 2D and 3D interactions of compound M5 and M6

**Structure activity relationship of Phenylcarbamoylpiperidine-1,2,4-triazole amide derivatives**

The previously synthesized derivatives^6^ were screened for identification of their *in-silico* inhibitory potential against NEK7 protein. A variation in docking scores from both software ranges from -22.88 to -31.38 kJ/mol. Compound M3, M5, M6, M7, M8 and M12 exhibited good binding energies with predicted inhibitory constant value ranges from 3.16 to 29.87 µM.

The 'R' substituent was shown to have a crucial role in defining free binding energies in the structural activity connection of compounds with docking scores from MOE and Autodock. Compound M12 had the highest docking energy in both MOE and Autodock softwares, with a predicted inhibitory constant value of 3.16 µM. On the nitrogen atom of compound M12, there is an aralkyl group with a dimethyl substituent in the ortho position. By giving an electron to the aryl ring, the methyl group produced a positive mesomeric action. The resonance effect of the aryl ring was modest, but it might be improved by substituting an electron donating group such as methyl. Furthermore, the substituent group's location had a significant impact on binding energy and the inhibitory constant value of a drug. The methyl group was replaced at ortho position on the benzene ring in compound M12, which increased its potency and selectivity. When M12 is compared to compound M8, which has an ethyl group at the para position of the benzene ring, the activity of M8 is marginally reduced, with a projected inhibitory constant of 10.69 µM. The replacement of an ethyl group at the para position resulted in a reduction in resonance. With a calculated inhibitory constant of 6.39 µM, compound M7 came in second. It has an aralkyl group on the nitrogen atom and an ortho position substitution of ethyl. The activity of the compound was boosted when the ortho position was substituted. When M7 is compared to the most potent compound M12, both have a substitution at the ortho position of the benzene ring, but the methyl substituent at the ortho position slightly increased the inhibitory potential of the compound because the methyl group donates electrons more readily than the ethyl group. It might be because the methyl group has a more favourable mesomeric impact. Compound M6 had a methyl group substituted at the meta position of the benzene ring, and its projected inhibitory constant value was 29.87 µM (Figure S7). The location of the substituent on the benzene ring was shown to be crucial in influencing the inhibitory potential of the molecule. When we compare compound M5 to the most potent compound M12, we can see that both have an aralkyl ring on the nitrogen atom, but M5 has only one methyl substituent at the ortho position of the benzene ring, whereas M12 has two methyl substituents at the ortho position, resulting in a greater mesomeric effect. Chemical M3 had a projected inhibition constant of 21.74 µM, which was owing to the cyclohexane ring being substituted on the nitrogen atom of the compound.

**Figure S7.** Structural activity relationship of potent derivatives

**Density Functional Theory**

**Global chemical reactivity descriptors**

HOMO LUMO energy values were used to calculate the global chemical reactivity descriptors such as hardness (η), chemical potential (µ), softness (S), electronegativity (χ) and electrophilicity index (ω) of the selected compounds. Small HOMO-LUMO energy gap corresponds to soft molecule having low kinetic stability and high chemical reactivity^7^. Chemical hardness values of compound M3, M7, M12 and dabrafenib in gas were 0.106eV, 0.097e, 0.099eV and 0.080 eV respectively. Whereas in solvent it were 0.104eV, 0.099eV, 0.100eV and 0.079 eV respectively (Table 4). These values indicate that potent derivatives have high chemical reactivity and low kinetic stability. The chemical reactivity of compounds differs with their structures. Chemical hardness and softness of compound **M4** (η=0.091 eV, S=5.51 eV) was best among all the compounds in gas phase. Thus, compound **M4** was assumed to be more reactive than all the compounds in gas phase. In solvent phase (methanol), compound **M12** was found to be more reactive as compared to all other compounds on the basis of values of hardness and softness (η=0.100 eV, S=5.01 eV). Compound **M7** in gas phase held higher electronegativity value (χ=0.121 eV) among all compounds so it would be the best electron acceptor. While in solvent phase (methanol), **M4** and **M6** shown the same value of higher electronegativity (χ=0.126 eV). So, both **M4** and **M6** would be the best electron acceptor among all compounds in solvent phase (methanol). Compound **M15** exhibited the higher value of electrophilicity index in gas phase as well as in solvent phase (methanol) (ω_gas_=0.087 eV, ω_solvent_=0.088 eV) indicates that it would be the stronger electrophiles among all compounds. Compound **M3** from gas phase while compound **M2** from solvent phase (methanol), both shown the smaller frontier orbital gap so, it would be more polarizable with high chemical reactivity, low kinetic stability and termed as soft molecule

**Table 1.** Quantum chemical descriptors of Triazole derivatives (M1-15) in gas and solvent phase.

| **Compound** | | **Hardness (η)** | **Softness (S)** | **Electronegativity (X)** | **Chemical Potential (μ)** | **Electrophilicity Index (ω)** |
| --- | --- | --- | --- | --- | --- | --- |
| M1 | Gas | 0.106 | 4.734 | 0.111 | -0.111 | 0.059 |
|  | Sol | 0.104 | 4.788 | 0.117 | -0.117 | 0.065 |
| M2 | Gas | 0.106 | 4.733 | 0.112 | -0.112 | 0.059 |
|  | Sol | 0.104 | 4.786 | 0.117 | -0.117 | 0.065 |
| M3 | Gas | 0.106 | 4.734 | 0.112 | -0.112 | 0.059 |
|  | Sol | 0.104 | 4.787 | 0.117 | -0.117 | 0.065 |
| M4 | Gas | 0.091 | 5.511 | 0.122 | -0.122 | 0.082 |
|  | Sol | 0.095 | 5.285 | 0.126 | -0.126 | 0.083 |
| M5 | Gas | 0.098 | 5.126 | 0.121 | -0.121 | 0.075 |
|  | Sol | 0.098 | 5.076 | 0.123 | -0.123 | 0.076 |
| M6 | Gas | 0.097 | 5.175 | 0.122 | -0.122 | 0.077 |
|  | Sol | 0.097 | 5.178 | 0.124 | -0.124 | 0.080 |
| M7 | Gas | 0.097 | 5.135 | 0.121 | -0.121 | 0.075 |
|  | Sol | 0.099 | 5.072 | 0.123 | -0.123 | 0.076 |
| M8 | Gas | 0.098 | 5.126 | 0.121 | -0.121 | 0.074 |
|  | Sol | 0.098 | 5.123 | 0.123 | -0.123 | 0.078 |
| M9 | Gas | 0.100 | 5.021 | 0.118 | -0.118 | 0.070 |
|  | Sol | 0.101 | 4.966 | 0.120 | -0.120 | 0.072 |
| M10 | Gas | 0.098 | 5.101 | 0.117 | -0.117 | 0.070 |
|  | Sol | 0.098 | 5.10 | 0.123 | -0.123 | 0.077 |
| M11 | Gas | 0.098 | 5.097 | 0.120 | -0.120 | 0.073 |
|  | Sol | 0.099 | 5.06 | 0.122 | -0.122 | 0.075 |
| M12 | Gas | 0.099 | 5.042 | 0.119 | -0.119 | 0.071 |
|  | Sol | 0.100 | 5.01 | 0.121 | -0.121 | 0.074 |
| M13 | Gas | 0.098 | 5.081 | 0.120 | -0.120 | 0.073 |
|  | Sol | 0.098 | 5.09 | 0.123 | -0.123 | 0.077 |
| M14 | Gas | 0.096 | 5.212 | 0.122 | -0.122 | 0.078 |
|  | Sol | 0.096 | 5.21 | 0.125 | -0.125 | 0.081 |
| M15 | Gas | 0.093 | 5.403 | 0.127 | -0.127 | 0.087 |
|  | Sol | 0.093 | 5.36 | 0.128 | -0.128 | 0.088 |
| dabrafenib | Gas | 0.080 | 6.28 | 0.154 | -0.154 | 0.149 |
|  | Sol | 0.079 | 6.34 | 0.152 | -0.152 | 0.147 |

Table 2 shows that compound, **M4** had the lowest energy gap of 0.181 eV in gas phase. Whereas, **M2** and **M3** showed the higher energy gap in gas phase and shown the same value of 0.211 eV. In solvent phase (methanol), **M15** had shown the smallest energy gap with value of 0.1866 eV. **M9** had slightly higher value than **M12** which was 0.2013 eV. Moreover, **M7, M8 and M12** had comparable energy gap differences. While in solvent phase (methanol), **M3** showed the highest energy gap of ∆E_gap_= 0.2088eV. The compound **M12** was with highest HOMO energy (E_HOMO_=-0.2181 eV) in gas phase. The highest HOMO energy in solvent phase (methanol) was shown by compound **M7** (E_HOMO_=-0.221 eV). The higher energy made them to be the best electron donors. The compound with the lowest LUMO energy in gas and solvent phases was **M1** i.e.**,** E_LUMO_=-0.00579 eV and E_LUMO_=-0.0121 eV respectively which predicted that it would be the best electron acceptor. The two parameters, ionization potential (I) and electron affinity (A) are related to HOMO and LUMO energies of compound respectively. The energy gap of HOMO-LUMO explains the electrical charge transfer within a molecule. The estimated of E_HOMO_, E_LUMO_, ΔE_gap_, A, I, η, µ, S, χ and ω of all derivatives are shown in Table 2.

**Table 2.** Energetic parameters of compounds (M1-15) in gas and solvent phase

| **Compound** | | **E_HOMO_**  **(eV)** | **E_LUMO_**  **(eV)** | **∆E_gap_**  **(eV)** | **Potential**  **Ionization I(eV)** | **Affinity A(eV)** | **Electron**  **donating power (ω-)** | **Electron**  **accepting Power (ω+)** | **Electrophilicity (Δω±)** |
| --- | --- | --- | --- | --- | --- | --- | --- | --- | --- |
| M1 | Gas | -0.217 | -0.005 | -0.211 | 0.217 | -0.005 | 0.117 | 0.011 | 0.128 |
|  | Sol | -0.221 | -0.012 | 0.208 | 0.221 | 0.012 | 0.136 | 0.020 | 0.156 |
| M2 | Gas | -0.217 | -0.006 | -0.211 | 0.217 | 0.006 | 0.128 | 0.016 | 0.145 |
|  | Sol | -0.221 | -0.012 | 0.208 | 0.221 | 0.012 | 0.136 | 0.020 | 0.156 |
| M3 | Gas | -0.217 | -0.005 | -0.211 | 0.217 | 0.005 | 0.128 | 0.016 | 0.144 |
|  | Sol | -0.221 | -0.012 | 0.208 | 0.221 | 0.012 | 0.136 | 0.020 | 0.156 |
| M4 | Gas | -0.212 | -0.031 | -0.181 | 0.212 | 0.031 | 0.154 | 0.032 | 0.186 |
|  | Sol | -0.220 | -0.031 | 0.189 | 0.220 | 0.031 | 0.158 | 0.032 | 0.191 |
| M5 | Gas | -0.218 | -0.023 | -0.195 | 0.218 | 0.023 | 0.147 | 0.026 | 0.174 |
|  | Sol | -0.221 | -0.024 | 0.196 | 0.221 | 0.024 | 0.150 | 0.027 | 0.177 |
| M6 | Gas | -0.218 | -0.025 | -0.193 | 0.218 | 0.025 | 0.149 | 0.028 | 0.177 |
|  | Sol | -0.221 | -0.027 | 0.193 | 0.221 | 0.027 | 0.155 | 0.030 | 0.185 |
| M7 | Gas | -0.218 | -0.023 | -0.194 | 0.218 | 0.023 | 0.147 | 0.027 | 0.174 |
|  | Sol | -0.221 | -0.023 | 0.197 | 0.221 | 0.023 | 0.150 | 0.027 | 0.177 |
| M8 | Gas | -0.218 | -0.022 | -0.195 | 0.218 | 0.022 | 0.147 | 0.026 | 0.173 |
|  | Sol | -0.220 | -0.025 | 0.195 | 0.220 | 0.025 | 0.152 | 0.028 | 0.180 |
| M9 | Gas | -0.217 | -0.018 | -0.199 | 0.217 | 0.018 | 0.142 | 0.023 | 0.165 |
|  | Sol | -0.221 | -0.019 | 0.201 | 0.221 | 0.019 | 0.145 | 0.024 | 0.169 |
| M10 | Gas | -0.214 | -0.018 | -0.196 | 0.214 | 0.018 | 0.140 | 0.024 | 0.164 |
|  | Sol | -0.220 | -0.024 | 0.196 | 0.220 | 0.024 | 0.151 | 0.028 | 0.179 |
| M11 | Gas | -0.217 | -0.021 | -0.196 | 0.217 | 0.021 | 0.145 | 0.026 | 0.171 |
|  | Sol | -0.221 | -0.023 | 0.197 | 0.221 | 0.023 | 0.149 | 0.027 | 0.176 |
| M12 | Gas | -0.218 | -0.019 | -0.198 | 0.218 | 0.019 | 0.143 | 0.024 | 0.168 |
|  | Sol | -0.221 | -0.021 | 0.199 | 0.221 | 0.021 | 0.147 | 0.026 | 0.172 |
| M13 | Gas | -0.217 | -0.021 | -0.196 | 0.217 | 0.021 | 0.145 | 0.025 | 0.170 |
|  | Sol | -0.221 | -0.024 | 0.196 | 0.221 | 0.024 | 0.150 | 0.028 | 0.178 |
| M14 | Gas | -0.218 | -0.026 | -0.191 | 0.218 | 0.026 | 0.151 | 0.029 | 0.180 |
|  | Sol | -0.220 | -0.028 | 0.192 | 0.220 | 0.028 | 0.156 | 0.031 | 0.186 |
| M15 | Gas | -0.219 | -0.034 | -0.185 | 0.219 | 0.034 | 0.161 | 0.035 | 0.196 |
|  | Sol | -0.221 | -0.034 | 0.186 | 0.221 | 0.034 | M15 | 0.163 | 0.035 |
| Dabrafenib | Gas | -0.233 | -0.074 | 0.159 | 0.233 | 0.074 | 0.236 | 0.082 | 0.318 |
|  | Sol | -0.231 | -0.073 | 0.158 | 0.231 | 0.073 | 0.233 | 0.081 | 0.313 |

**ADMET properties**

Furthermore, it was discovered that all compounds had an ideal value for caco-2 permeability that was greater than -5.01. The Caco-2 permeability cell line is a human colon epithelial cell line that correlates with improved human intestine absorption. Compounds with the highest Caco-2+ permeability have a greater intestine absorption rate. In terms of carcinogenicity, all chemicals have the potential to cause cancer. The observed AMES toxicity profile values revealed that all substances had a chance of being AMES hazardous. The synthetic accessibility score (SA score) is used to measure the ease with which drug-like molecules may be synthesised, and it was discovered that all substances have a positive SA score. All compounds had a superior ADMET profile in general; all values are listed in Table 3.

**Table 3.** Detailed ADMET properties of all compounds

| **ABSORPTION & DISTRIBUTION PROPERTIES** | | | | | | | | |
| --- | --- | --- | --- | --- | --- | --- | --- | --- |
|  | **VOLUME OF DISTRIBUTION (VD)** | **HUMAN INTESTINAL ABSORPTION (HIA)** | **CACO-2 PERMEABILITY** | **BLOOD BRAIN BARRIER (BBB) & BLOOD-PLACENTA BARRIER (BPB** | **PLASMA PROTEIN BINDING (PPB)** | **PGP-INHIBITOR** | **P-GLYCOPROTEIN SUBSTRATE (PGP-SUBSTRATE)** | **MDCK PERMEABILITY** |
| M1 | 1.46 | 0.271 | -5.319 | 0.884 | 93.50% | 0.004 | 0.998 | 6e-06 |
| M2 | 1.553 | 0.212 | -5.386 | 0.944 | 92.75% | 0.056 | 0.999 | 5e-06 |
| M3 | 1.3 | 0.044 | -5.296 | 0.641 | 95.26% | 0.091 | 0.999 | 1.2e-05 |
| M4 | 1.065 | 0.497 | -5.472 | 0.754 | 96.37% | 0.935 | 0.995 | 1.1e-05 |
| M5 | 1.09 | 0.02 | -5.279 | 0.487 | 97.51% | 0.991 | 0.992 | 1.6e-05 |
| M6 | 1.134 | 0.049 | -5.47 | 0.637 | 97.78% | 0.997 | 0.995 | 1.1e-05 |
| M7 | 1.177 | 0.637 | -5.216 | 0.412 | 97.80% | 0.998 | 0.995 | 2e-05 |
| M8 | 1.192 | 0.048 | -5.365 | 0.35 | 97.93% | 0.999 | 0.997 | 1.1e-05 |
| M9 | 1.083 | 0.011 | -5.24 | 0.39 | 97.99% | 0.99 | 0.99 | 1.4e-05 |
| M10 | 1.03 | 0.01 | -5.171 | 0.446 | 97.91% | 0.998 | 0.99 | 1.2e-05 |
| M11 | 1.069 | 0.01 | -5.185 | 0.442 | 98.07% | 0.998 | 0.991 | 1.2e-05 |
| M12 | 1.024 | 0.009 | -5.011 | 0.416 | 98.04% | 0.994 | 0.981 | 1.7e-05 |
| M13 | 1.185 | 0.015 | -5.446 | 0.526 | 98.26% | 0.998 | 0.995 | 9e-06 |
| M14 | 1.183 | 0.534 | -5.519 | 0.636 | 97.13% | 0.937 | 0.995 | 1.2e-05 |
| M15 | 1.069 | 0.01 | -5.185 | 0.422 | 98.07% | 0.998 | 0.991 | 1.2e-05 |
| **METABOLISM** | | | | | | | **EXCRETION** | |
|  | **CYP1A2 inhibitor** | **CYP2C19**  **inhibitor** | **CYP2C9 inhibitor** | **CYP2D6 inhibitor** | **CYP3A4 inhibitor** | | **CL** | **T1/2** |
| M1 | 0.017 | 0.355 | 0.055 | 0.119 | 0.242 | | 8.844 | 0.449 |
| M2 | 0.028 | 0.456 | 0.274 | 0.189 | 0.652 | | 6.71 | 0.588 |
| M3 | 0.028 | 0.675 | 0.657 | 0.189 | 0.892 | | 4.813 | 0.25 |
| M4 | 0.045 | 0.755 | 0.694 | 0.261 | 0.907 | | 5.699 | 0.427 |
| M5 | 0.05 | 0.758 | 0.834 | 0.303 | 0.912 | | 4.717 | 0.435 |
| M6 | 0.074 | 0.866 | 0.89 | 0.543 | 0.93 | | 5.477 | 0.337 |
| M7 | 0.072 | 0.839 | 0.893 | 0.482 | 0.919 | | 4.673 | 0.385 |
| M8 | 0.067 | 0.844 | 0.925 | 0.627 | 0.912 | | 5.276 | 0.26 |
| M9 | 0.05 | 0.795 | 0.87 | 0.42 | 0.927 | | 4.992 | 0.327 |
| M10 | 0.047 | 0.756 | 0.855 | 0.303 | 0.927 | | 4.978 | 0.371 |
| M11 | 0.054 | 0.793 | 0.878 | 0.406 | 0.935 | | 4.902 | 0.306 |
| M12 | 0.03 | 0.594 | 0.801 | 0.228 | 0.933 | | 4.324 | 0.493 |
| M13 | 0.06 | 0.812 | 0.898 | 0.545 | 0.933 | | 5.565 | 0.217 |
| M14 | 0.061 | 0.79 | 0.852 | 0.394 | 0.866 | | 5.242 | 0.411 |
| M15 | 0.054 | 0.793 | 0.878 | 0.406 | 0.935 | | 4.902 | 0.306 |
| **MEDICINAL PROPERTIES** | | | **TOXICITY** | | | | | |
|  | **Synthetic Accessibility Score** | **Lipinski Rule** | **AMES Toxicity** | **Carcinogenicity** | **Eye Corrosion** | **Eye Irritation** | **Respiratory Toxicity** | |
|  |  |  |  |  |  |  |  | |
| M1 | 2.364 | Accepted | 0.028 | 0.773 | 0.003 | 0.014 | 0.643 | |
| M2 | 2.256 | Accepted | 0.024 | 0.657 | 0.003 | 0.012 | 0.54 | |
| M3 | 2.347 | Accepted | 0.03 | 0.634 | 0.003 | 0.011 | 0.714 | |
| M4 | 2.232 | Accepted | 0.038 | 0.449 | 0.003 | 0.01 | 0.36 | |
| M5 | 2.239 | Accepted | 0.164 | 0.829 | 0.003 | 0.013 | 0.746 | |
| M6 | 2.262 | Accepted | 0.092 | 0.761 | 0.003 | 0.012 | 0.654 | |
| M7 | 2.316 | Accepted | 0.109 | 0.782 | 0.003 | 0.011 | 0.709 | |
| M8 | 2.274 | Accepted | 0.091 | 0.836 | 0.003 | 0.011 | 0.533 | |
| M9 | 2.321 | Accepted | 0.286 | 0.826 | 0.003 | 0.012 | 0.684 | |
| M10 | 2.303 | Accepted | 0.28 | 0.848 | 0.003 | 0.012 | 0.717 | |
| M11 | 2.311 | Accepted | 0.186 | 0.823 | 0.003 | 0.011 | 0.712 | |
| M12 | 2.341 | Accepted | 0.235 | 0.847 | 0.003 | 0.011 | 0.758 | |
| M13 | 2.305 | Accepted | 0.212 | 0.806 | 0.003 | 0.013 | 0.63 | |
| M14 | 2.182 | Accepted | 0.076 | 0.772 | 0.003 | 0.014 | 0.765 | |
| M15 | 2.228 | Accepted | 0.186 | 0.823 | 0.003 | 0.011 | 0.712 | |
| **TOX21 PATHWAY** | | | | | | | | |
|  | **NR-AR** | | **NR-AR-LBD** | | **NR-ER** | | **Antioxidant Response Element** | |
|  |  | |  | |  | |  | |
| M1 | 0.21 | | 0.037 | | 0.116 | | 0.595 | |
| M2 | 0.443 | | 0.013 | | 0.18 | | 0.607 | |
| M3 | 0.341 | | 0.026 | | 0.23 | | 0.71 | |
| M4 | 0.644 | | 0.07 | | 0.183 | | 0.645 | |
| M5 | 0.614 | | 0.08 | | 0.193 | | 0.688 | |
| M6 | 0.563 | | 0.041 | | 0.244 | | 0.725 | |
| M7 | 0.534 | | 0.115 | | 0.212 | | 0.72 | |
| M8 | 0.403 | | 0.08 | | 0.263 | | 0.798 | |
| M9 | 0.643 | | 0.027 | | 0.176 | | 0.764 | |
| M10 | 0.611 | | 0.022 | | 0.206 | | 0.687 | |
| M11 | 0.594 | | 0.028 | | 0.208 | | 0.707 | |
| M12 | 0.678 | | 0.013 | | 0.139 | | 0.617 | |
| M13 | 0.598 | | 0.029 | | 0.231 | | 0.744 | |
| M14 | 0.551 | | 0.143 | | 0.223 | | 0.709 | |
| M15 | 0.594 | | 0.028 | | 0.208 | | 0.707 | |

**Molecular Dynamic Simulations**

**Evaluation of structural stability, compactness, and energies**

The analysis of the stability of systems was further studied by examining the energies, radius of gyration (R_g_), and solvent accessible surface area (SASA). The PME (Particle mesh Ewald) algorithm was used to compute the interactions resulted from electrostatic forces such as Coulomb and Van der Waals interactions^14^. During this procedure, a cutoff of 1.5 nm was chosen for the Coulomb and van der Waals interactions, so as to eliminate potential trouble and errors in the short and long-time cuts. The error range in real space was set to 1 × 10^-8^, which corresponds to the parameter K (k=0.020822755 nm)^15^. Radius of gyration is the mass-weighted RMS distance of the collection of atoms from their common centre of mass^8^. R_g_ is a critical marker in analysing the stability of proteins and its complexes in MD simulation studies^9^. The R_g_ of NEK7 alone and NEK7-M12 complex is shown in **Figure S8 (A)**. There was some fluctuation in R_g_ of both system during initial phases of simulation, but it became consent after 50 ns, showing that the system became well equilibrated after 50 ns. The average R_g_ of NEK7 and NEK7-M12 complex was found to be 1.930 and 1.955 nm, respectively. The R_g_ trajectory remained almost uniform in entire simulation illustrating the stability of both systems in aqueous environment. The negligible fluctuation in R_g_ also shows that there were insignificant conformational changes in NEK7 and its complex during simulation^10^. SASA is another useful tool to study the structural compactness of proteins during MD simulation^11^. SASA of NEK7 and NEK7-M12 complex as function of time is shown in **Figure S8 (B)**.

**
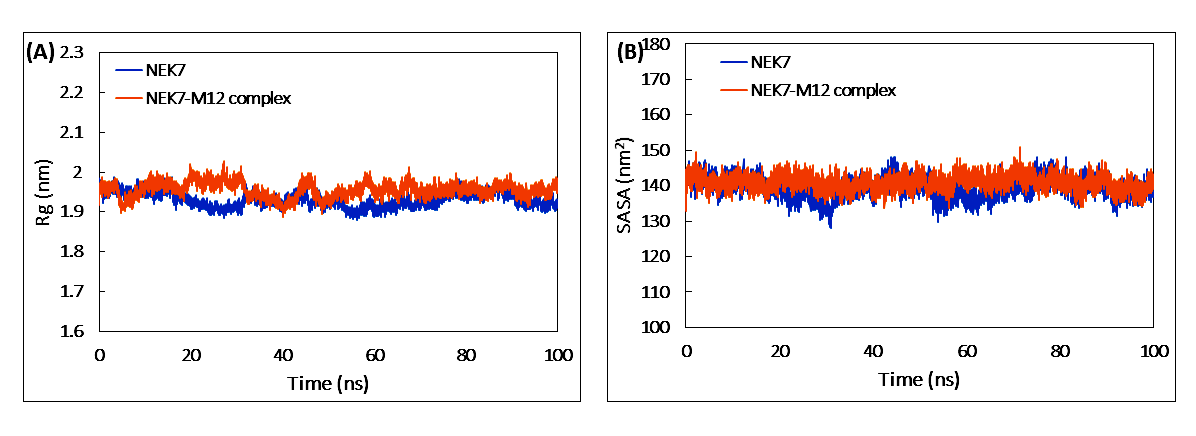
**

**Figure 8.** (A) Radius of gyration (Rg) of NEK7 and NEK7-M12 complex as function of time. (B) Solvent accessible surface area (SASA) of NEK7 and NEK7-M12 complex as function of time.

The average SASA of NEK7 and NEK7-M12 complex was obtained as 139.374 and 140.954 nm^2^, respectively. The analysis of SASA further shows that binding of M12 with NEK7 did exert any noticeable effect on the compactness of the protein

**Analysis of hydrogen bonds and assessment of secondary structure**

The binding of M12 with NEK7 was examined by calculating the number of hydrogen bonds and hydrogen bond profile. The hydrogen bond profile with more than 1% existence is shown in **Figure S9 (A)**. As evident from the data, there was continuous existence of hydrogen bonds during simulation. The number of hydrogen bonds as function of time is shown in **Figure S9 (B)**.

**
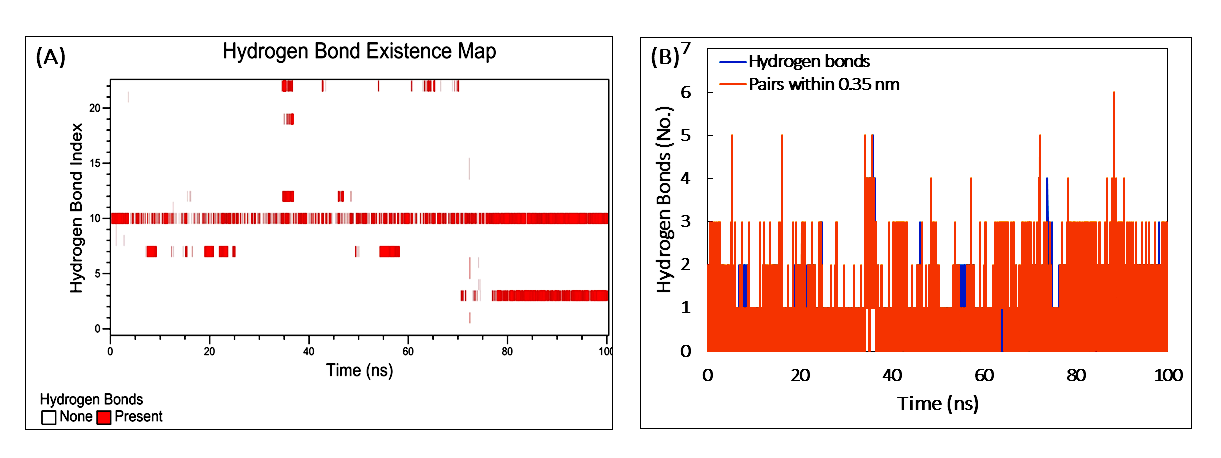
**

**Figure S9.** (A) Hydrogen bonds existence map for interaction of M12 with NEK7 during MD simulation. (B) Number of hydrogen binds formed between M12 and NEK7 during MD simulation.

The average number of hydrogen bond was nearly which shows that there was at least one hydrogen bond between M12 and NEK7 during entire simulation time. The effect of binding of M12 on the secondary structural stability of NEK7 was also examined and the result is shown in **Figure S10**. The average percentage of coils, β-sheets, β-bridges, bends, turns, α-helices, and 3-helices in NEK alone were found to be 23.25, 17.04, 0.56, 6.25, 14.45, 34.01, and 4.41, respectively. Similarly, the average content of coils, β-sheets, β-bridges, bends, turns, α-helices, and 3-helices in NEK-M12 complex was 22.78, 16.27, 0.58, 6.80, 13.52, 34.42, and 5.59%, respectively. The data clearly shows that there were negligible changes in the secondary structural motifs of NEK7 after interaction of M12. The finding shows the structural stability of the NEK7 and its complex with M12 the aqueous medium^12^.

**
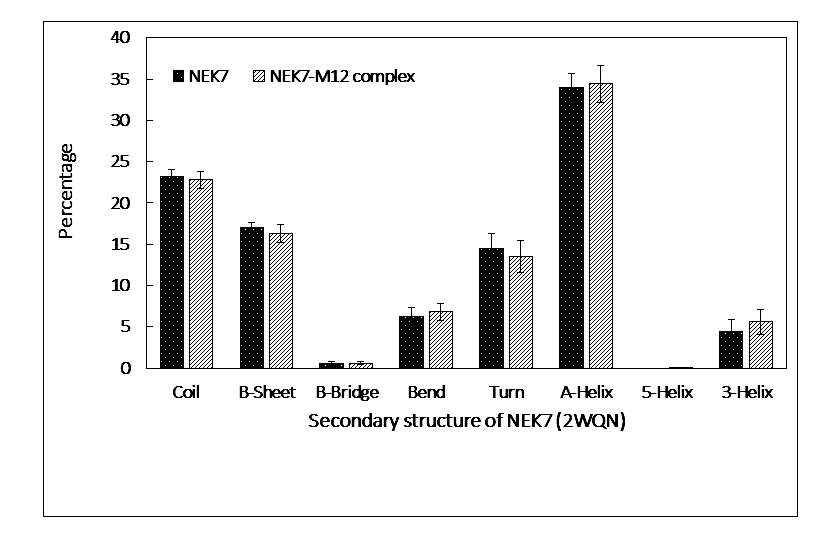
**

**Figure S10.** Average secondary structural components of NEK7 in the absence and presence of M12.

**Analysis of principal component and essential dynamics**

Principal component analysis (PCA) is a statistical procedure to assess the collective motion in biological macromolecules during molecular dynamic simulations. PCA is performed by reducing the dimension of dataset keeping the critical information which is characterized by the eigenvectors^13^. PCA was performed to examine the ﬂexibility in NEK7 in the absence and presence of M12. The projection of eigenvectors of NEK7 alone and NEK7-M12 complex is shown in **Figure S11**.


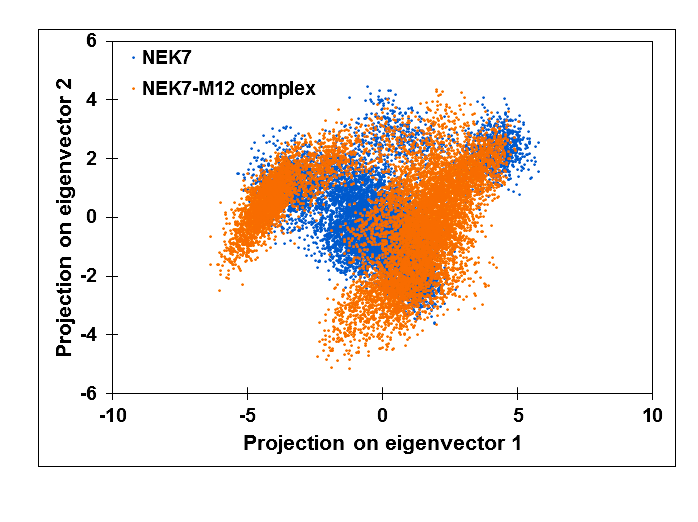


**Figure S11.** 2-D scatter plot of principal component analysis (PCA) by projecting the eigenvectors of NEK7 and NEK7-M12 complex

Both NEK7 alone and its complex occupied similar confirmational space in 2D projection. The free energy landscape of the NEK7 and its complex was plotted to analyse the structural conformations and obtain global energy minima basins. The free energy landscape of NEK7 in absence and presence of M12 is shown in **Figure S12 (A)**. The landscapes shows both systems reached their respective energy minima. The 3D structures corresponding to the lowest energy point in the energy minima basin was extracted from the respective trajectories. The Ramachandran plots of energy minima structures of NEK7 and NEK7-M12 complex is shown in **Figure S12 (B)**. No residue was found in the disallowed region for both NEK7 alone and NEK7-M12 complex. The Ramachandran plots further validates the secondary structural data in which there was negligible effect on secondary structure of the protein after interaction of M12.

**
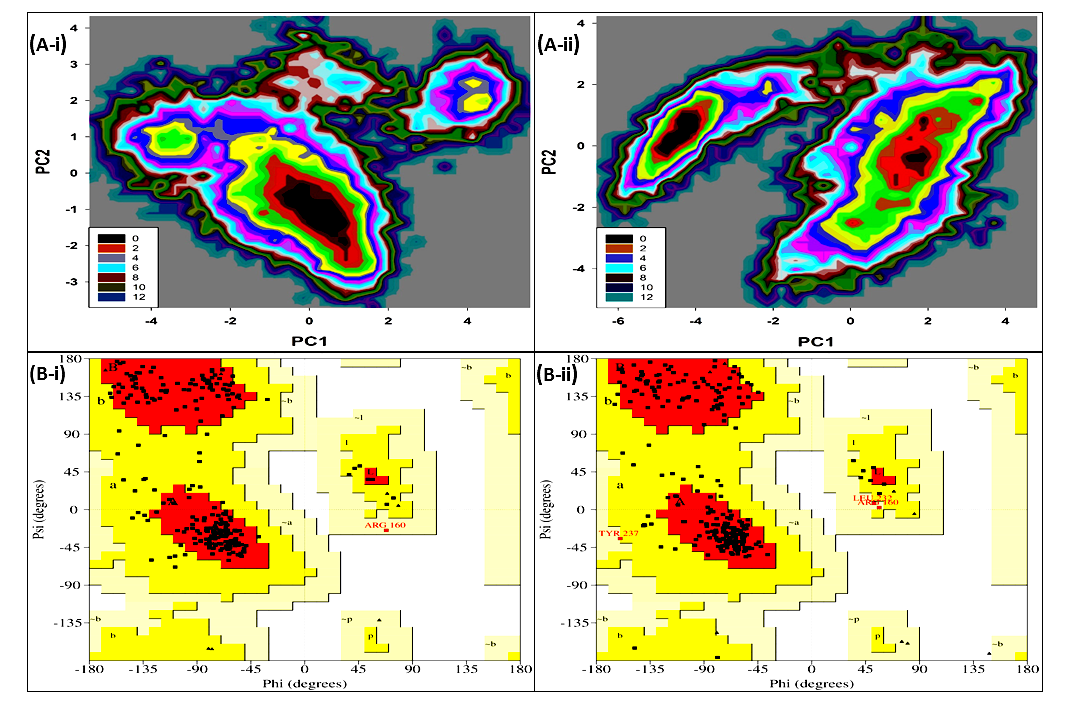
**

**Figure S12.** (A-i) Free energy landscape plot of NEK7. (A-ii) Free energy landscape plot of NEK7-M12 complex. (B-i) Ramachandran plot of NEK7. (B-ii) Ramachandran plot of NEK7-M12 complex.

**References**

1 Byrne, M. J. *et al.* Nek7 conformational flexibility and inhibitor binding probed through protein engineering of the R-spine. **477**, 1525-1539 (2020).

2 Kornev, A. P., Taylor, S. S. J. B. E. B. A.-P. & Proteomics. Defining the conserved internal architecture of a protein kinase. **1804**, 440-444 (2010).

3 Richards, M. W. *et al.* An autoinhibitory tyrosine motif in the cell-cycle-regulated Nek7 kinase is released through binding of Nek9. **36**, 560-570 (2009).

4 Minoguchi, S., Minoguchi, M., Yoshimura, A. J. B. & communications, b. r. Differential control of the NIMA-related kinases, Nek6 and Nek7, by serum stimulation. **301**, 899-906 (2003).

5 Johnson, L. N., Noble, M. E. & Owen, D. J. J. C. Active and inactive protein kinases: structural basis for regulation. **85**, 149-158 (1996).

6 Muzaffar, S. *et al.* Evaluation of Ethylated Phenylcarbamoylazinane‐1, 2, 4‐Triazole Amides Derivatives as 15‐Lipoxygenase Inhibitors Together with Cytotoxic, ADME and Molecular Modeling Studies. **5**, 14210-14216 (2020).

7 Azarakhshi, F., Khaleghian, M. & Farhadyar, N. J. L. i. O. C. DFT study and NBO analysis of conformational properties of 2-Substituted 2-Oxo-1, 3, 2-dioxaphosphorinanes and their dithia and diselena analogs. **12**, 516-522 (2015).

8 Qais, F. A. *et al.* Plumbagin inhibits quorum sensing-regulated virulence and biofilms of Gram-negative bacteria: in vitro and in silico investigations. **37**, 724-739 (2021).

9 Qais, F. A. *et al.* Glyburide inhibits non-enzymatic glycation of HSA: An approach for the management of AGEs associated diabetic complications. **169**, 143-152 (2021).

10 Rath, B. *et al.* Design, synthesis and molecular modeling studies of novel mesalamine linked coumarin for treatment of inflammatory bowel disease. **41**, 128029 (2021).

11 Ahmad, S. *et al.* A comparative study based on activity, conformation and computational analysis on the inhibition of human salivary aldehyde dehydrogenase by phthalate plasticizers: Implications in assessing the safety of packaged food items. **462**, 152947 (2021).

12 Qais, F. A. *et al.* Coumarin exhibits broad-spectrum antibiofilm and antiquorum sensing activity against gram-negative bacteria: in vitro and in silico investigation. **6**, 18823-18835 (2021).

13 Siddiqui, S. *et al.* Biophysical insight into the binding mechanism of doxofylline to bovine serum albumin: An in vitro and in silico approach. **249**, 119296 (2021).

14 Cheatham, T. E. I.; Miller, J. L.; Fox, T.; Darden, T. A.; Kollman, P. A. Molecular Dynamics Simulations on Solvated Biomolecular Systems: The Particle Mesh Ewald Method Leads to Stable Trajectories of DNA, RNA, and Proteins https://pubs.acs.org/doi/pdf/10.1021/ja00119a045 (accessed 2022 -02 -15). https://doi.org/10.1021/ja00119a045.

15 Shirts, M. R.; Klein, C.; Swails, J. M.; Yin, J.; Gilson, M. K.; Mobley, D. L.; Case, D. A.; Zhong, E. D. Lessons Learned from Comparing Molecular Dynamics Engines on the SAMPL5 Dataset. *J. Comput. Aided Mol. Des.* **2017**, *31* (1), 147–161. https://doi.org/10.1007/s10822-016-9977-1.
